# Correction: Unravelling the Structural and Molecular Basis Responsible for the Anti-Biofilm Activity of Zosteric Acid

**DOI:** 10.1371/journal.pone.0136124

**Published:** 2015-08-14

**Authors:** The PLOS ONE Staff

## Notice of Republication

This article was republished on July 21, 2015 to correct errors in the figure order that were introduced during the typesetting process. The publisher apologizes for the errors. Please download this article again to view the correct version. The originally published, uncorrected article and the republished, corrected article are provided here for reference.

## Supporting Information

S1 File Originally published, uncorrected article. (PDF)

S2 File Republished, corrected article. (PDF)
