# Supplementary material for: Unravelling the Structural and Molecular Basis Responsible for the Anti-Biofilm Activity of Zosteric Acid
Source: PLoS One. 2015 Jul 1;10(7):e0131519. doi: 10.1371/journal.pone.0131519 (PMC4488431; doi:10.1371/journal.pone.0131519)
Supplement: S1 Protocol — (PDF) [file pone.0131519.s001.pdf]

S1 Protocol. General procedure for the synthesis of compounds 2, 7, 8, 11, 17, 23, 24, 33, 34, 36 -38.

General procedure for the synthesis of substituted cinnamic acids (2, 7, 8, 11, 17) [1].

To a solution of the suitable substituted benzaldehyde (1 mmol) in pyridine (1 mL), malonic acid (2.20 mmol) and piperidine (0.2 mmol) were added. The reaction mixture was stirred under reflux for 2 hours and then cooled to room temperature. The solution was poured into 2N ice-cold aqueous HCl (2 mL) and the precipitate was collected by filtration, washed with water and dried under vacuum.

4-methylcinnamic acid (2). Yield: 70 %. White solid. ^{1}H NMR (DMSO): 2.29 (s, 3H, CH_3), 6.45 (d, 1H, CH, J = 15.9), 7.19 (d, 2H, ArH, J = 7.8), 7.53 (d, 2H, ArH, J = 7.8), 7.54 (d, 1H, CH, J = 15.9), 12.33 (br s, 1H, COOH). ^{13}C NMR (DMSO): 21.7, 118.8, 128.8, 130.2, 132.2, 140.8, 144.6, 168.4.

4-cyanocinnamic acid (7). Yield: 60 %. White solid. ^{1}H NMR (DMSO): 6.70 (d, 1H, CH, J = 16.2), 7.62 (d, 1H, CH, J = 16.2), 7.83-7.90 (m, 4H, ArH). ^{13}C NMR (DMSO): 112.8, 119.3, 123.5, 129.5, 133.4, 139.5, 142.6, 167.8.

4-carboxycinnamic acid (8). Yield: 75 %. White solid.^{1}H NMR (DMSO): 6.62 (d, 1H, CH, J = 15.9), 7.62 (d, 1H, CH, J = 15.9), 7.78 (d, 2H, ArH, J = 8.1), 7.93 (d, 2H, ArH, J = 8.1). ^{13}C NMR (DMSO): 122.5, 128.9, 130.4, 132.5, 139.0, 143.3, 167.5, 168.0.

4-trifluoromethylcinnamic acid (11). Yield: 66 %. White solid. ^{1}H NMR (DMSO): 6.67 (d, 1H, CH, J = 15.9), 7.65 (d, 1H, CH, J = 15.9), 7.75 (d, 2H, ArH, J = 8.4), 7.91 (d, 2H, ArH, J = 8.4). ^{19}F NMR (DMSO): - 62 (s, 3F).^{13}C NMR (DMSO): 122.8, 126.3, 129.5, 130.7, 139.0, 142.7, 167.8.

2-chlorocinnamic acid (17). Yield: 50 %. White solid. ^{1}H NMR ($CDCl_3$): 6.46 (d, 1H, CH, J = 15.9), 7.27-7.38 (m, 2H, ArH), 7.44 (dd, 1H, ArH, J = 1.5, J = 6.9), 7.65 (dd, 1H, ArH, J = 1.5, J = 6.9), 8.22 (d, 1H, CH, J = 15.9). ^{13}C NMR (DMSO): 123.0, 128.5, 129.0, 130.6, 132.4, 132.5, 134.2, 139.4, 167.8.

Synthesis of *cis* ethyl cinnamate (36) [2].

To a stirred solution of the commercially available ethyl phenylpropiolate (300 mg, 1.72 mmol) in methanol (4 mL) were added Lindlar catalyst (5 % Pd on $CaCO_3$ poisned with lead) and pyridine (0.15 mL, 1.72 mmol). The reaction mixture was hydrogenated at room temperature for 3 hours. At the end of the reaction, the catalyst was filtered off through a Celite pad and the solvent was concentrated under reduced pressure. The crude was purified by column chromatography (cyclohexane/ethyl acetate 95:5) to afford compound **36**.

Yield: 51 %. Colourless oil. ^{1}H NMR ($CDCl_3$): 1.25 (t, 3H, CH_3, J = 7.2), 4.19 (q, 2H, CH_2, J = 7.2, J = 14.1), 5.97 (d, 1H, CH, J = 12.6), 6.96 (d, 1H, CH, J = 12.6), 7.34-7.36 (m, 3H, ArH), 7.58-7.59 (m, 2H, ArH). ^{13}C NMR ($CDCl_3$): 14.4, 60.5, 120.1, 128.2, 129.2, 130.0, 135.1, 143.3, 166.5.

Synthesis of *cis* cinnamic acid (37) [2].

To a stirred solution of *cis* ethyl cinnamate **36** (100 mg, 0.57 mmol) in ethanol (1 mL) and tetrahydrofuran (1 mL), 1N aqueous solution of NaOH (1 mL) was added. The solution was stirred at room temperature for 12 hours. The reaction was monitored by TLC until completion, then the solvent was evaporated and water (2 mL) was added. The aqueous layer was acidified to pH = 1 with 2N HCl and then extracted with ethyl acetate (3 x 1 mL). The combined organic layers were washed with brine, dried over anhydrous sodium sulfate and the solvent was evaporated under reduced pressure. The crude was purified by flash chromatography eluting by dichloromethane/methanol (95:5) to afford compound **37**.

Yield: 93 %. Colourless oil. ^{1}H NMR ($CDCl_3$): 5.98 (d, 1H, CH, J = 12.6), 7.08 (d, 1H, CH, J = 12.6), 7.36-7.39 (m, 3H, ArH), 7.60-7.64 (m, 2H, ArH). ^{13}C NMR ($CDCl_3$): 118.9, 128.3, 129.6, 130.2, 134.6, 145.9, 170.1.

Synthesis of cis zosteric acid sodium salt (38)

In a microwave vessel, to a solution of *cis* coumaric acid **32** (100 mg, 0.61 mmol) in dry acetonitrile (1.5 mL), sulphur trioxide pyridine complex Py·SO_3 (194 mg, 1.22 mmol) was added under an inert atmosphere. The resulting mixture was irradiated in a microwave synthesizer at 300 Watts at the temperature of 120°C for 25 minutes. After cooling, the solvent was evaporated under reduced pressure. Then 1N NaOH was added dropwise to pH = 7. The resulting mixture was extracted with dichloromethane (3 x 2 mL) and the aqueous phase was evaporated to dryness. The residue was treated with methanol (5 mL) and the undissolved material filtered out. The evaporation of solvent provided compound **38.**

Yield: 50 %. Brown solid. ^{1}H NMR (CD_3OD): 6.44 (d, 1H, CH, J = 12.4), 7.30 (d, 2H, ArH, J = 8.8), 7.44 (d, 1H, CH, J = 12.4), 7.52 (d, 2H, ArH, J = 8.8). ^{13}C NMR (DMSO): 121.1, 125.3, 128.7, 131.2, 139..0, 154.8, 170.9. Mass spectrometry: electron spray ionization technique ESI-MS (+) *m/z* 289.

General procedure for the esterification of cinnamic acid derivatives (23, 24, 33) [3].

The suitable cinnamic acid (1 mmol) was dissolved in methanol (1 mL) and 96 % H_2SO_4 (0.13 mL) was added dropwise. The obtained solution was refluxed for 1 hour. Then, the solvent was removed, the residue was diluted with ethyl acetate (1 mL), washed with water (1 x 2 mL) and a saturated solution of sodium hydrogencarbonate (1 x 2 mL). The organic layer was dried over anhydrous Na_2SO_4 and concentrated *in vacuo*.

***trans* methyl coumarate (23).** Quantitative yield. White solid. ^{1}H NMR ($CDCl_3$): 3.81 (s, 3H, CH_3), 6.30 (d, 1H, CH, J = 16.2), 6.87 (d, 2H, ArH, J = 8.7), 7.41 (d, 2H, ArH, J = 8.7), 7.65 (d, 1H, CH, J = 16.2). ^{13}C NMR ($CDCl_3$): 55.12, 114.9, 116.2, 127.0, 130.3, 145.4, 158.5, 168.8.

***trans* methyl 4-methoxycinnamate (24).** Quantitative yield. White solid. ^{1}H NMR ($CDCl_3$): 3.80 (s, 3H, CH_3), 3.84 (s, 3H, CH_3), 6.31 (d, 1H, CH, J = 16.2), 6.90 (d, 2H, ArH, J = 8.7), 7.47 (d, 2H,

ArH, J = 8.7), 7.65 (d, 1H, CH, J = 16.2). ^{13}C NMR ($CDCl_3$): 51.5, 55.8, 114.0, 115.2, 127.1, 130.0, 145.3, 161.7, 169.1.

***cis* methyl coumarate (33).** Quantitative yield. White solid. ^{1}H NMR ($CDCl_3$): 3.80 (s, 3H, CH_3), 5.32 (br s, 1H, OH), 6.30 (d, 1H, CH, J = 16.2), 6.85 (d, 2H, ArH, J = 8.5), 7.43 (d, 2H, ArH, J = 8.5), 7.64 (d, 1H, CH, J = 16.2). ^{13}C NMR (DMSO): 51.9, 114.6, 116.4, 125.7, 131.0, 145.4, 160.5, 167.7.

Synthesis of *cis* methyl 4-methoxycinnamate (35) [3].

To a solution of the required methyl coumarate **33** (500 mg, 2.80 mmol) in dry *N,N*-dimethylformamide (1.5 mL), anhydrous potassium carbonate (580 mg, 4.20 mmol) and iodomethane (0.26 mL, 4.20 mmol) were added under a nitrogen atmosphere. The reaction mixture was stirred at reflux for 1.5 hours. After the evaporation of *N,N*-dimethylformamide, the resultant residue was extracted with ethyl acetate (3 x 2 mL) and washed with brine (1 x 2 mL). The collected organic phase was dried over anhydrous sodium sulfate, filtrated and the solvent was evaporated under reduced pressure. The crude was purified by flash chromatography (cyclohexane/ethyl acetate 7:3) to provide compound **35.**

Yield: 94 %. White solid. ^{1}H NMR ($CDCl_3$): 3.79 (s, 3H, CH_3), 3.83 (s, 3H, CH_3), 6.31 (d, 1H, CH, J = 16.2), 6.90 (d, 2H, ArH, J = 8.7), 7.47 (d, 2H, ArH, J = 8.7), 7.65 (d, 1H, CH, J = 16.2). ^{13}C NMR ($CDCl_3$): 51.5, 55.8, 114.0, 115.2, 127.1, 130.0, 145.3, 161.7, 169.1.

Synthesis of *cis* 4-methoxycinnamic acid (34)

To a stirred solution of *cis* methyl 4-methoxy cinnamate **35** (500 mg, 2.60 mmol) in ethanol (1 mL), 1N aqueous solution of NaOH (1 mL) was added. The solution was stirred at reflux for 2 hours. The reaction was monitored by TLC until completion, then the solvent was evaporated and water (2 mL) was added. The aqueous layer was acidified to pH = 1 with 2N HCl and then extracted with ethyl acetate (3 x 1 mL). The combined organic layers were washed with brine, dried over anhydrous

sodium sulfate and the solvent was evaporated under reduced pressure. The crude was purified by flash chromatography eluiting with dichloromethane/methanol (9:1) to afford the desired product **34.**

Quantitative yield. White solid. ^{1}H NMR ($CDCl_3$): 3.85 (s, 3H, CH_3), 6.33 (d, 1H, CH, J = 16.2), 6.93 (d, 2H, ArH, J = 8.5), 7.51 (d, 2H, ArH, J = 8.5), 7.75 (d, 1H, CH, J = 16.2). ^{13}C NMR ($CDCl_3$): 115.2, 126.4, 130.5, 147.1, 162.1, 172.9.

References

1. Szymanski W, Wu B, Weiner B, de Wildeman S, Feringa BL, Janssen DB. Phenylalanine aminomutase-catalyzed addition of ammonia to substituted cinnamic acids: a route to enantiopure alpha- and beta-amino acids. J Org Chem. 2009;74: 9152-9157.

2. Ueda S, Okada T, Nagasawa H. Oxindole synthesis by palladium-catalysed aromatic C-H alkenylation. Chem Commun. 2010;46: 2462-2464.

3. De P, Koumba Yoya G, Constant P, Bedos-Belval F, Duran H, Saffon N, et al. Design, synthesis, and biological evaluation of new cinnamic derivatives as antituberculosis agents. J Med Chem. 2011;54: 1449-1461.
