# Supplementary material for: Unravelling the Structural and Molecular Basis Responsible for the Anti-Biofilm Activity of Zosteric Acid
Source: PLoS One. 2015 Jul 1;10(7):e0131519. doi: 10.1371/journal.pone.0131519 (PMC4488431; doi:10.1371/journal.pone.0131519)
Supplement: S1 Table — Percentage reduction respect to the negative control is calculated as (ZA-related compound data—control data) x 100 / control data. a) Percentage reduction of the maximum specific growth rate: code (0) x >- 10%; code (- 1)- 10% ≤ x < -20%; code (- 2)- 20% ≤ x < -30%; code (- 3) x ≤ - 30%; b) Percentage reduction of the number of adhered cells: code (+ 1) x > + 20%; code (0) + 20% ≤ x < -20%; code (- 1)- 20% ≤ x < -30%; code (- 2)- 30% ≤ x < -40%; code (- 3) x ≤ - 40%. According to post hoc analysis (Tukey’s HSD, p < 0.05), data sharing the same letter indicate no significant difference. (PDF) [file pone.0131519.s002.pdf]

S1A Table. Planktonic growth and cell adhesion in the presence of ZA and compounds 1-6.

		Planktonic growth			Cell adhesion		
**Cmpd**	**Conc. (µM)**	**A_{600}/min (x10^{-3})**	**Reduction (%)**	**Code (a)**	**N. adhered cells (x10^{7})**	**Reduction (%)**	**Code (b)**
ZA	0	1.28±0.03^{a}	0.00	0	1.92±0.18^{b}	0.00	0
	0.183	1.32±0.06^{a}	2.86	0	2.77±0.17^{d}	44.59	1
	1.83	1.31±0.04^{a}	1.76	0	1.54±0.29^{c}	-19.76	0
	18.3	1.29±0.04^{a}	0.11	0	1.55±0.19^{c}	-19.34	0
	183	1.30±0.04^{a}	1.07	0	1.39±0.13^{c}	-27.67	-1
	1830	1.30±0.04^{a}	1.21	0	0.56±0.12^{a}	-70.56	-3
1	0	1.28±0.03^{abc}	0.00	0	1.92±0.18^{b}	0.00	0
	0.183	1.31±0.03^{ab}	2.06	0	2.29±0.16^{b}	19.37	0
	1.83	1.26±0.04^{abc}	-1.72	0	1.03±0.14^{ce}	-46.05	-3
	18.3	1.23±0.02^{abc}	-3.99	0	1.42±0.24^{de}	-25.80	-1
	183	1.23±0.06^{abc}	-3.87	0	1.49±0.14^{de}	-22.36	-1
	1830	0.97±0.04^{e}	-24.17	-2	1.22±0.27^{cde}	-36.38	-2
2	0	1.28±0.03^{a}	0.00	0	1.92±0.18^{bc}	0.00	0
	0.183	1.33±0.03^{a}	3.41	0	1.86±0.23^{bcd}	-3.15	0
	1.83	1.32±0.03^{a}	2.66	0	0.47±0.09^{a}	-75.40	-3
	18.3	1.26±0.07^{a}	-2.23	0	1.50±0.18^{cde}	-21.94	-1
	183	1.28±0.07^{a}	-0.48	0	1.39±0.32^{de}	-27.44	-1
	1830	0.87±0.03^{b}	-32.54	-3	1.40±0.28^{de}	-27.01	-1
3	0	1.28±0.03^{abc}	0.00	0	1.92±0.18^{b}	0.00	0
	0.183	1.23±0.06^{abd}	-4.42	0	1.26±0.15^{ce}	-34.41	-1
	1.83	1.33±0.03^{ac}	3.46	0	0.73±0.07^{ade}	-61.92	-3
	18.3	1.32±0.03^{ac}	3.08	0	1.04±0.23^{cde}	-45.97	-3
	183	1.28±0.05^{abc}	-0.03	0	1.17±0.28^{ce}	-39.12	-2
	1830	1.02±0.01^{e}	-20.44	-2	1.33±0.08^{ce}	-30.69	-2
4	0	1.28±0.03^{abc}	0.00	0	1.92±0.18^{bef}	0.00	0
	0.183	1.24±0.04^{abcd}	-3.32	0	1.25±0.43^{cdef}	-34.95	-2
	1.83	1.22±0.01^{abcdf}	-4.74	0	1.17±0.17^{cdf}	-38.77	-2
	18.3	1.19±0.04^{bcdef}	-7.58	0	1.78±0.10^{bcef}	-7.37	0
	183	1.14±0.05^{def}	-11.37	-1	1.44±0.15^{bcdef}	-25.03	-1
	1830	0.83±0.02^{h}	-35.10	-3	1.32±0.28^{cdef}	-31.11	-2
5	0	1.28±0.03^{abc}	0.00	0	1.92±0.18^{bceh}	0.00	0
	0.183	1.24±0.02^{ac}	-3.14	0	1.92±0.26^{bcefgh}	0.37	0
	1.83	1.23±0.03^{ac}	-4.12	0	1.17±0.11^{defl}	-39.15	-2
	18.3	1.14±0.01^{df}	-11.53	-1	1.58±0.13^{bcdefghl}	-17.47	0
	183	1.15±0.06^{de}	-10.43	-1	1.36±0.24^{bcdefghl}	-28.79	-1
	1830	0.94±0.04^{g}	-26.65	-2	1.14±0.25^{df}	-40.52	-3
6	0	1.28±0.03^{a}	0.00	0	1.92±0.18^{b}	0.00	0
	0.183	1.30±0.07^{a}	1.21	0	2.30±0.62^{b}	19.90	0
	1.83	1.25±0.05^{a}	-2.36	0	2.05±0.20^{b}	7.00	0
	18.3	1.31±0.01^{a}	2.38	0	1.12±0.21^{c}	-41.44	-3
	183	1.16±0.03^{b}	-10.03	-1	1.25±0.25^{c}	-34.97	-2
	1830	0.85±0.03^{d}	-33.69	-3	1.39±0.23^{c}	-27.46	-1

Percentage reduction respect to the negative control is calculated as (ZA-related compound data – control data) x 100 / control data. a) Percentage reduction of the maximum specific growth rate: code (0) x > -10 %; code (-1) -10 % ≤ x < -20 %; code (-2) -20 % ≤ x < -30 %; code (-3) x ≤ -30 %; b) Percentage reduction of the number of adhered cells: code (+1) x > +20 %; code (0) +20 % ≤ x < -20 %; code (-1) -20 % ≤ x < -30 %; code (-2) -30 % ≤ x < -40 %; code (-3) x ≤ -40 %. According to post hoc analysis (Tukey's HSD, $p < 0.05$), data sharing the same letter indicate no significant difference.

S1B Table. Planktonic growth and cell adhesion in the presence of compounds 7-13.

Cmpd	Conc. (μM)	Planktonic growth: A_{600}/min (x10^{-3})	Planktonic growth: Reduction (%)	Planktonic growth: Code (a)	Cell adhesion: N. adhered cells (x10^7)	Cell adhesion: Reduction (%)	Cell adhesion: Code (b)
7	0	1.28±0.03[a]	0.00	0	1.92±0.18[bc]	0.00	0
	0.183	1.29±0.03[a]	0.12	0	1.73±0.32[bcd]	-9.57	0
	1.83	1.26±0.02[a]	-1.99	0	1.75±0.37[bc]	-8.60	0
	18.3	1.31±0.00[a]	1.90	0	1.88±0.41[bc]	-1.95	0
	183	1.28±0.05[a]	-0.41	0	1.56±0.20[bcd]	-18.67	0
	1830	0.96±0.04[c]	-24.88	-2	1.18±0.23[cd]	-38.63	-2
8	0	1.28±0.03[a]	0.00	0	1.92±0.18[b]	0.00	0
	0.183	1.30±0.04[a]	1.13	0	1.63±0.37[b]	-14.90	0
	1.83	1.26±0.05[a]	-1.68	0	1.14±0.19[c]	-40.28	-3
	18.3	1.27±0.03[a]	-1.14	0	1.06±0.18[c]	-44.92	-3
	183	1.28±0.05[a]	-0.51	0	1.14±0.11[c]	-40.73	-3
	1830	1.11±0.01[b]	-13.24	-1	2.19±0.28[d]	14.29	0
9	0	1.28±0.03[a]	0.00	0	1.92±0.18[bc]	0.00	0
	0.183	1.25±0.04[a]	-2.79	0	1.75±0.21[bcd]	-8.89	0
	1.83	1.27±0.03[a]	-0.79	0	1.43±0.25[cde]	-25.63	-1
	18.3	1.30±0.05[a]	1.06	0	1.18±0.31[de]	-38.64	-2
	183	1.12±0.00[b]	-12.86	-1	1.40±0.31[cde]	-27.19	-1
	1830	0.83±0.02[c]	-35.27	-3	1.14±0.22[de]	-40.36	-3
10	0	1.28±0.03[a]	0.00	0	1.92±0.18[bc]	0.00	0
	0.183	1.26±0.04[a]	-1.86	0	1.81±0.18[bcdf]	-5.41	0
	1.83	1.24±0.02[a]	-3.08	0	1.47±0.17[cdef]	-23.51	-1
	18.3	1.24±0.02[a]	-3.14	0	1.19±0.22[deg]	-37.94	-2
	183	1.25±0.06[a]	-2.43	0	1.57±0.18[cdf]	-18.09	0
	1830	0.87±0.02[d]	-32.40	-3	1.10±0.28[deg]	-42.38	-3
11	0	1.28±0.03[abc]	0.00	0	1.92±0.18[b]	0.00	0
	0.183	1.25±0.04[abc]	-2.93	0	2.26±0.16[c]	18.06	0
	1.83	1.24±0.02[abc]	-3.19	0	1.53±0.17[d]	-20.05	-1
	18.3	1.31±0.01[ab]	2.04	0	1.43±0.28[d]	-25.44	-1
	183	1.24±0.04[ac]	-3.38	0	0.40±0.06[a]	-79.23	-3
	1830	0.87±0.01[f]	-32.56	-3	0.48±0.03[a]	-74.86	-3
12	0	1.28±0.03[ab]	0.00	0	1.92±0.18[bc]	0.00	0
	0.183	1.34±0.07[ab]	4.38	0	1.82±0.44[bcd]	-4.88	0
	1.83	1.29±0.05[abc]	0.19	0	1.82±0.11[bcd]	-5.16	0
	18.3	1.22±0.05[bc]	-4.95	0	1.63±0.29[bcd]	-14.70	0
	183	1.21±0.03[c]	-5.56	0	1.54±0.30[cd]	-19.83	0
	1830	0.99±0.03[d]	-23.12	-2	1.30±0.33[d]	-32.21	-2
13	0	1.28±0.03[ac]	0.00	0	1.92±0.18[bc]	0.00	0
	0.183	1.29±0.02[ac]	0.41	0	1.78±0.05[bce]	-7.13	0
	1.83	1.22±0.08[ab]	-4.66	0	1.75±0.19[bce]	-8.79	0
	18.3	1.31±0.02[ac]	2.40	0	1.31±0.24[cdef]	-31.41	-2
	183	1.25±0.03[abc]	-2.47	0	1.59±0.18[de]	-17.14	0
	1830	1.00±0.02[d]	-22.39	-2	1.19±0.24[df]	-37.93	-2

Percentage reduction respect to the negative control is calculated as (ZA-related compound data – control data) x 100 / control data. a) Percentage reduction of the maximum specific growth rate: code (0) x > -10 %; code (-1) -10 % ≤ x < -20 %; code (-2) -20 % ≤ x < -30 %; code (-3) x ≤ -30 %; b) Percentage reduction of the number of adhered cells: code (+1) x > +20 %; code (0) +20 % ≤ x < -20 %; code (-1) -20 % ≤ x < -30 %; code (-2) -30 % ≤ x < -40 %; code (-3) x ≤ -40 %. According to post hoc analysis (Tukey's HSD, p < 0.05), data sharing the same letter indicate no significant difference.

S1C Table. Planktonic growth and cell adhesion in the presence of compounds 14-20.

		Planktonic growth			Cell adhesion		
**Cmpd**	**Conc. (µM)**	**A_{600}/min (x10^{-3})**	**Reduction (%)**	**Code (a)**	**N. adhered cells (x10^{7})**	**Reduction (%)**	**Code (b)**
14	0	1.28 ± 0.03^{abc}	0.00	0	1.92 ± 0.18^{bcef}	0.00	0
	0.183	1.21 ± 0.02^{abcde}	-5.68	0	1.93 ± 0.06^{bcefg}	0.68	0
	1.83	1.27 ± 0.01^{abc}	-1.20	0	1.70 ± 0.14^{bcef}	-11.20	0
	18.3	1.23 ± 0.04^{abcd}	-3.89	0	1.33 ± 0.21^{abcef}	-30.56	-2
	183	1.30 ± 0.02^{abc}	1.57	0	2.29 ± 0.50^{bcef}	19.62	0
	1830	1.14 ± 0.03^{bde}	-10.95	-1	4.25 ± 1.35^{hi}	121.57	+
15	0	1.28 ± 0.03^{a}	0.00	0	1.92 ± 0.18^{bc}	0.00	0
	0.183	1.31 ± 0.05^{a}	1.90	0	1.76 ± 0.08^{bce}	-7.98	0
	1.83	1.27 ± 0.05^{a}	-1.23	0	1.72 ± 0.18^{bce}	-10.36	0
	18.3	1.30 ± 0.01^{a}	1.46	0	1.41 ± 0.14^{de}	-26.16	-1
	183	1.29 ± 0.03^{a}	0.63	0	1.53 ± 0.23^{cde}	-19.96	0
	1830	0.86 ± 0.02^{b}	-32.67	-3	1.58 ± 0.26^{cde}	-17.47	0
16	0	1.28 ± 0.03^{a}	0.00	0	1.92 ± 0.18^{bf}	0.00	0
	0.183	1.29 ± 0.04^{a}	0.21	0	3.59 ± 0.37^{c}	87.42	+
	1.83	1.31 ± 0.05^{a}	2.05	0	1.46 ± 0.19^{def}	-23.68	-1
	18.3	1.28 ± 0.04^{a}	-0.29	0	1.36 ± 0.19^{de}	-28.82	-1
	183	1.29 ± 0.05^{a}	0.13	0	1.74 ± 0.11^{bdf}	-8.95	0
	1830	1.03 ± 0.05^{d}	-20.16	-2	1.33 ± 0.17^{de}	-30.57	-2
17	0	1.28 ± 0.03^{abc}	0.00	0	1.92 ± 0.18^{bd}	0.00	0
	0.183	1.28 ± 0.03^{abcd}	-0.29	0	3.62 ± 0.37^{c}	88.79	+
	1.83	1.30 ± 0.07^{abcd}	0.87	0	1.76 ± 0.08^{bdf}	-8.09	0
	18.3	1.33 ± 0.04^{ab}	3.62	0	1.37 ± 0.14^{ef}	-28.33	-1
	183	1.29 ± 0.02^{abc}	0.68	0	1.49 ± 0.19^{def}	-22.26	-1
	1830	1.05 ± 0.04^{ef}	-17.95	-1	1.08 ± 0.15^{h}	-43.68	-3
18	0	1.28 ± 0.03^{ab}	0.00	0	1.92 ± 0.18^{bcd}	0.00	0
	0.183	1.28 ± 0.02^{ab}	0.07	0	2.16 ± 0.15^{bc}	12.75	0
	1.83	1.26 ± 0.04^{abc}	-1.68	0	1.89 ± 0.19^{bcd}	-1.22	0
	18.3	1.31 ± 0.04^{ab}	1.79	0	1.61 ± 0.23^{bd}	-16.05	0
	183	1.20 ± 0.03^{bc}	-6.82	0	1.95 ± 0.25^{bcd}	1.50	0
	1830	1.00 ± 0.05^{c}	-22.04	-2	2.79 ± 0.46^{e}	45.65	+
19	0	1.28 ± 0.03^{a}	0.00	0	1.92 ± 0.18^{bc}	0.00	0
	0.183	1.30 ± 0.06^{a}	0.88	0	1.93 ± 0.44^{bcd}	0.81	0
	1.83	1.29 ± 0.05^{a}	0.50	0	1.67 ± 0.12^{bcd}	-12.94	0
	18.3	1.30 ± 0.01^{a}	1.58	0	1.65 ± 0.19^{cd}	-13.94	0
	183	1.32 ± 0.04^{a}	2.69	0	1.80 ± 0.28^{bcd}	-6.18	0
	1830	1.05 ± 0.04^{b}	-18.13	-1	1.61 ± 0.14^{cd}	-15.75	0
20	0	1.28 ± 0.03^{a}	0.00	0	1.92 ± 0.18^{bc}	0.00	0
	0.183	1.30 ± 0.03^{a}	1.40	0	1.91 ± 0.33^{bcd}	-0.13	0
	1.83	1.31 ± 0.03^{a}	2.18	0	1.58 ± 0.10^{cde}	-17.44	0
	18.3	1.30 ± 0.04^{a}	1.14	0	1.52 ± 0.23^{de}	-20.60	-1
	183	1.29 ± 0.04^{a}	0.12	0	0.60 ± 0.04^{a}	-68.90	-3
	1830	1.13 ± 0.02^{b}	-11.61	-1	0.72 ± 0.13^{a}	-62.34	-3

Percentage reduction respect to the negative control is calculated as (ZA-related compound data – control data) x 100 / control data. a) Percentage reduction of the maximum specific growth rate: code (0) x > -10 %; code (-1) -10 % ≤ x < -20 %; code (-2) -20 % ≤ x < -30 %; code (-3) x ≤ -30 %; b) Percentage reduction of the number of adhered cells: code (+1) x > +20 %; code (0) +20 % ≤ x < -20 %; code (-1) -20 % ≤ x < -30 %; code (-2) -30 % ≤ x < -40 %; code (-3) x ≤ -40 %. According to post hoc analysis (Tukey's HSD, $p < 0.05$), data sharing the same letter indicate no significant difference.

S1D Table. Planktonic growth and cell adhesion in the presence of compounds 21-27.

Cmpd	Conc. (μM)	Planktonic growth: A_{600}/min (x10^{-3})	Planktonic growth: Reduction (%)	Planktonic growth: Code (a)	Cell adhesion: N. adhered cells (x10^{7})	Cell adhesion: Reduction (%)	Cell adhesion: Code (b)
21	0	1.28±0.03[a]	0.00	0	1.92±0.18[bc]	0.00	0
	0.183	1.32±0.04[a]	2.92	0	1.71±0.16[bcd]	-10.57	0
	1.83	1.30±0.01[a]	0.95	0	1.60±0.18[cde]	-16.42	0
	18.3	1.30±0.05[a]	1.62	0	1.72±0.23[bcd]	-10.51	0
	183	1.32±0.00[a]	2.52	0	1.34±0.18[def]	-30.22	-2
	1830	1.03±0.04[b]	-20.07	-2	1.24±0.16[def]	-35.24	-2
22	0	1.28±0.03[acd]	0.00	0	1.92±0.18[b]	0.00	0
	0.183	1.21±0.01[bcdf]	-5.58	0	3.07±0.17[c]	60.29	+
	1.83	1.29±0.03[acd]	0.75	0	2.66±0.23[ce]	38.91	+
	18.3	1.29±0.02[acd]	0.43	0	1.37±0.06[deg]	-28.42	-1
	183	1.24±0.01[abcdf]	-3.61	0	1.18±0.33[df]	-38.53	-2
	1830	1.17±0.06[bcf]	-9.14	0	0.99±0.04[degh]	-48.14	-3
23	0	1.28±0.03[a]	0.00	0	1.92±0.18[b]	0.00	0
	0.183	1.32±0.02[a]	2.93	0	2.26±0.22[c]	17.80	0
	1.83	1.29±0.07[a]	0.37	0	1.22±0.15[d]	-36.25	-2
	18.3	1.31±0.06[a]	2.26	0	1.50±0.16[d]	-21.75	-1
	183	1.12±0.03[b]	-12.66	-1	1.24±0.15[d]	-35.24	-2
	1830	0.56±0.02[c]	-56.28	-3	1.47±0.23[d]	-23.17	-1
24	0	1.28±0.03[a]	0.00	0	1.92±0.18[b]	0.00	0
	0.183	1.26±0.03[a]	-1.69	0	4.77±0.47[c]	148.78	+
	1.83	1.29±0.01[a]	0.37	0	1.83±0.22[b]	-4.76	0
	18.3	1.31±0.01[a]	2.32	0	1.96±0.12[b]	2.38	0
	183	1.15±0.03[b]	-10.36	-1	4.16±0.54[c]	117.10	+
	1830	1.09±0.01[b]	-15.26	-1	9.52±1.19[e]	396.86	+
25	0	1.28±0.03[a]	0.00	0	1.92±0.18[b]	0.00	0
	0.183	1.27±0.01[a]	-0.72	0	2.60±0.15[c]	35.59	+
	1.83	1.29±0.02[a]	0.83	0	1.73±0.16[b]	-9.71	0
	18.3	1.24±0.05[a]	-3.48	0	1.67±0.08[b]	-12.95	0
	183	1.12±0.04[b]	-12.58	-1	1.64±0.24[b]	-14.33	0
	1830	0.55±0.01[c]	-56.89	-3	1.12±0.15[d]	-41.78	-3
26	0	1.28±0.03[ab]	0.00	0	1.92±0.18[bc]	0.00	0
	0.183	1.31±0.02[ab]	2.18	0	1.63±0.13[bcd]	-15.20	0
	1.83	1.27±0.04[abc]	-1.37	0	1.58±0.17[cd]	-17.76	0
	18.3	1.26±0.06[abc]	-1.92	0	1.52±0.25[cd]	-20.80	-1
	183	1.25±0.03[abc]	-2.92	0	1.47±0.11[cd]	-23.44	-1
	1830	0.81±0.03[e]	-37.06	-3	1.62±0.20[cd]	-15.63	0
27	0	1.28±0.03[a]	0.00	0	1.92±0.18[bde]	0.00	0
	0.183	1.28±0.04[a]	-0.57	0	2.52±0.45[c]	31.74	+
	1.83	1.29±0.06[a]	0.23	0	1.99±0.23[bd]	4.07	0
	18.3	1.29±0.05[a]	0.20	0	1.95±0.11[bd]	1.79	0
	183	1.29±0.02[a]	0.60	0	1.67±0.18[be]	-12.90	0
	1830	1.15±0.03[b]	-10.65	-1	1.71±0.16[be]	-10.97	0

Percentage reduction respect to the negative control is calculated as (ZA-related compound data – control data) x 100 / control data. a) Percentage reduction of the maximum specific growth rate: code (0) $x > -10$ %; code (-1) $-10 \% \leq x < -20$ %; code (-2) $-20 \% \leq x < -30$ %; code (-3) $x \leq -30$ %; b) Percentage reduction of the number of adhered cells: code (+1) $x > +20$ %; code (0) $+20 \% \leq x < -20$ %; code (-1) $-20 \% \leq x < -30$ %; code (-2) $-30 \% \leq x < -40$ %; code (-3) $x \leq -40$ %. According to post hoc analysis (Tukey's HSD, $p < 0.05$), data sharing the same letter indicate no significant difference.

S1E Table. Planktonic growth and cell adhesion in the presence of compounds 28-34.

		Planktonic growth			Cell adhesion		
**Cmpd**	**Conc. (μM)**	**A_{600}/min ($x10^{-3}$)**	**Reduction (%)**	**Code (a)**	**N. adhered cells ($x10^{7}$)**	**Reduction (%)**	**Code (b)**
28	0	1.28 ± 0.03^{a}	0.00	0	1.92 ± 0.18^{b}	0.00	0
	0.183	1.31 ± 0.02^{a}	1.67	0	1.94 ± 0.01^{b}	1.10	0
	1.83	1.28 ± 0.02^{a}	-0.20	0	1.99 ± 0.15^{b}	3.77	0
	18.3	1.28 ± 0.03^{a}	-0.61	0	1.76 ± 0.10^{b}	-8.15	0
	183	1.28 ± 0.05^{a}	0.01	0	1.26 ± 0.18^{c}	-34.39	-2
	1830	0.86 ± 0.02^{c}	-33.01	-3	0.92 ± 0.14^{d}	-52.00	-3
29	0	1.28 ± 0.03^{a}	0.00	0	1.92 ± 0.18^{bd}	0.00	0
	0.183	1.29 ± 0.05^{a}	0.29	0	2.56 ± 0.26^{c}	33.75	1
	1.83	1.29 ± 0.04^{a}	0.23	0	1.79 ± 0.11^{bd}	-6.83	0
	18.3	1.31 ± 0.06^{a}	1.72	0	1.93 ± 0.24^{bd}	0.73	0
	183	1.28 ± 0.05^{a}	-0.43	0	2.14 ± 0.15^{bcd}	11.51	0
	1830	0.76 ± 0.02^{b}	-40.52	-3	1.42 ± 0.27^{d}	-25.78	-1
30	0	1.28 ± 0.03^{a}	0.00	0	1.92 ± 0.18^{be}	0.00	0
	0.183	1.23 ± 0.01^{a}	-3.95	0	2.38 ± 0.21^{ce}	24.26	1
	1.83	1.29 ± 0.02^{a}	0.31	0	1.82 ± 0.18^{be}	-5.29	0
	18.3	1.32 ± 0.03^{a}	2.86	0	1.97 ± 0.19^{be}	2.99	0
	183	1.26 ± 0.05^{a}	-2.10	0	2.06 ± 0.22^{bce}	7.33	0
	1830	0.83 ± 0.03^{b}	-35.51	-3	1.40 ± 0.16^{f}	-26.98	-1
31	0	1.28 ± 0.03^{a}	0.00	0	1.92 ± 0.18^{b}	0.00	0
	0.183	1.23 ± 0.03^{a}	-3.94	0	2.85 ± 0.43^{c}	48.69	1
	1.83	1.26 ± 0.00^{a}	-2.14	0	1.58 ± 0.29^{b}	-17.68	0
	18.3	1.29 ± 0.01^{a}	0.61	0	1.99 ± 0.43^{b}	4.02	0
	183	1.24 ± 0.04^{a}	-3.25	0	1.85 ± 0.22^{b}	-3.23	0
	1830	0.82 ± 0.02^{d}	-36.19	-3	0.94 ± 0.19^{e}	-50.79	-3
32	0	1.28 ± 0.03^{a}	0.00	0	1.92 ± 0.18^{bc}	0.00	0
	0.183	1.29 ± 0.07^{a}	0.30	0	1.39 ± 0.15^{abc}	-27.34	-1
	1.83	1.27 ± 0.05^{a}	-0.88	0	1.64 ± 0.11^{bc}	-14.39	0
	18.3	1.27 ± 0.04^{a}	-1.26	0	1.83 ± 0.14^{bc}	-4.30	0
	183	1.28 ± 0.06^{a}	-0.16	0	1.62 ± 0.19^{bc}	-15.26	0
	1830	1.25 ± 0.02^{a}	-2.25	0	5.45 ± 1.00^{e}	184.36	1
33	0	1.28 ± 0.03^{a}	0.00	0	1.92 ± 0.18^{b}	0.00	0
	0.183	1.25 ± 0.06^{a}	-2.75	0	3.90 ± 0.27^{c}	103.54	1
	1.83	1.26 ± 0.03^{a}	-1.49	0	1.88 ± 0.09^{b}	-1.99	0
	18.3	1.32 ± 0.02^{a}	2.61	0	1.45 ± 0.19^{d}	-24.08	-1
	183	1.13 ± 0.03^{b}	-12.31	-1	1.31 ± 0.24^{d}	-31.43	-2
	1830	0.74 ± 0.02^{de}	-42.25	-3	1.51 ± 0.16^{d}	-21.25	-1
34	0	1.28 ± 0.03^{a}	0.00	0	1.92 ± 0.18^{b}	0.00	0
	0.183	1.26 ± 0.03^{a}	-2.19	0	5.75 ± 0.32^{e}	199.95	1
	1.83	1.28 ± 0.05^{a}	0.08	0	1.93 ± 0.16^{bc}	0.83	0
	18.3	1.29 ± 0.04^{a}	0.64	0	1.14 ± 0.09^{bc}	-40.43	-3
	183	1.10 ± 0.02^{b}	-14.41	-1	3.02 ± 0.12^{d}	57.49	1
	1830	1.00 ± 0.04^{c}	-22.27	-2	2.64 ± 0.31^{d}	37.76	1

Percentage reduction respect to the negative control is calculated as (ZA-related compound data – control data) x 100 / control data. a) Percentage reduction of the maximum specific growth rate: code (0) $x > -10$ %; code (-1) $-10\ \% \leq x < -20$ %; code (-2) $-20\ \% \leq x < -30$ %; code (-3) $x \leq -30$ %; b) Percentage reduction of the number of adhered cells: code (+1) $x > +20$ %; code (0) $+20\ \% \leq x < -20$ %; code (-1) $-20\ \% \leq x < -30$ %; code (-2) $-30\ \% \leq x < -40$ %; code (-3) $x \leq -40$ %. According to post hoc analysis (Tukey's HSD, $p < 0.05$), data sharing the same letter indicate no significant difference.

S1F Table. Planktonic growth and cell adhesion in the presence of compounds 35-41.

		Planktonic growth			Cell adhesion		
**Cmpd**	**Conc. (µM)**	**A_{600}/min ($x10^{-3}$)**	**Reduction (%)**	**Code (a)**	**N. adhered cells ($x10^{7}$)**	**Reduction (%)**	**Code (b)**
35	0	1.28±0.03^{a}	0.00	0	1.92±0.18^{bcd}	0.00	0
	0.183	1.26±0.05^{a}	-2.19	0	2.87±0.56^{bc}	49.69	1
	1.83	1.25±0.07^{a}	-2.61	0	1.72±0.19^{bd}	-10.13	0
	18.3	1.16±0.03^{b}	-9.96	0	1.12±0.14^{e}	-41.80	-3
	183	0.92±0.05^{c}	-28.33	-2	2.85±0.31^{f}	48.83	1
	1830	0.55±0.02^{d}	-56.93	-3	5.72±0.25^{h}	198.51	1
36	0	1.28±0.03^{ab}	0.00	0	1.92±0.18^{bdf}	0.00	0
	0.183	1.23±0.02^{abc}	-4.03	0	2.87±0.56^{c}	49.69	1
	1.83	1.24±0.02^{abc}	-3.79	0	1.78±0.24^{bd}	-7.38	0
	18.3	1.21±0.03^{bcd}	-5.71	0	1.13±0.17^{e}	-40.87	-3
	183	1.15±0.04^{cd}	-10.75	-1	2.27±0.17^{bf}	18.70	0
	1830	1.08±0.02^{d}	-15.57	-1	5.60±0.65^{h}	192.07	1
37	0	1.28±0.03^{ab}	0.00	0	1.92±0.18^{b}	0.00	0
	0.183	1.32±0.04^{ab}	2.71	0	2.46±0.17^{b}	28.52	1
	1.83	1.28±0.07^{ab}	-0.56	0	2.36±0.26^{b}	23.00	1
	18.3	1.30±0.03^{ab}	1.27	0	1.35±0.28^{b}	-29.61	-1
	183	1.24±0.05^{a}	-3.17	0	5.08±0.53^{c}	164.82	1
	1830	0.88±0.02^{c}	-31.64	-3	5.43±0.76^{c}	183.29	1
38	0	1.28±0.03^{a}	0.00	0	1.92±0.18^{bd}	0.00	0
	0.183	1.31±0.13^{a}	2.28	0	3.79±0.61^{c}	98.02	1
	1.83	1.31±0.01^{a}	1.66	0	3.69±0.41^{c}	92.49	1
	18.3	1.31±0.03^{a}	2.24	0	3.54±0.20^{c}	84.50	1
	183	1.24±0.03^{a}	-3.39	0	2.11±0.14^{bd}	9.90	0
	1830	1.22±0.03^{a}	-4.98	0	4.30±1.01^{c}	124.16	1
39	0	1.28±0.03^{a}	0.00	0	1.92±0.18^{b}	0.00	0
	0.183	1.29±0.03^{a}	0.31	0	1.86±0.35^{b}	-3.07	0
	1.83	1.27±0.04^{a}	-0.89	0	1.94±0.34^{b}	1.04	0
	18.3	1.26±0.06^{a}	-2.16	0	1.98±0.24^{b}	3.58	0
	183	1.27±0.02^{a}	-1.39	0	2.10±0.50^{b}	9.41	0
	1830	0.95±0.05^{b}	-25.66	-2	1.83±0.19^{b}	-4.37	0
40	0	1.28±0.03^{a}	0.00	0	1.92±0.18^{bd}	0.00	0
	0.183	1.26±0.04^{a}	-2.04	0	3.21±0.39^{cd}	67.46	1
	1.83	1.24±0.03^{a}	-3.05	0	1.90±0.20^{bcd}	-0.85	0
	18.3	1.29±0.05^{a}	0.56	0	1.92±0.16^{bcd}	0.24	0
	183	1.31±0.03^{a}	2.23	0	1.93±0.28^{bcd}	0.60	0
	1830	0.82±0.03^{b}	-36.31	-3	1.39±0.24^{bd}	-27.57	-1
41	0	1.28±0.03^{ac}	0.00	0	1.92±0.18^{b}	0.00	0
	0.183	1.27±0.04^{abc}	-0.89	0	2.09±0.40^{b}	9.15	0
	1.83	1.24±0.04^{ab}	-3.19	0	1.63±0.21^{d}	-15.16	0
	18.3	1.24±0.03^{ab}	-3.45	0	1.87±0.20^{bd}	-2.50	0
	183	1.33±0.03^{ac}	3.88	0	1.13±0.38^{ae}	-41.15	-3
	1830	1.16±0.03^{d}	-9.88	0	0.70±0.08^{ae}	-63.30	-3

Percentage reduction respect to the negative control is calculated as (ZA-related compound data – control data) x 100 / control data. a) Percentage reduction of the maximum specific growth rate: code (0) x > -10 %; code (-1) -10 % ≤ x < -20 %; code (-2) -20 % ≤ x < -30 %; code (-3) x ≤ -30 %; b) Percentage reduction of the number of adhered cells: code (+1) x > +20 %; code (0) +20 % ≤ x < -20 %; code (-1) -20 % ≤ x < -30 %; code (-2) -30 % ≤ x < -40 %; code (-3) x ≤ -40 %. According to post hoc analysis (Tukey's HSD, $p < 0.05$), data sharing the same letter indicate no significant difference.

S1G Table. Planktonic growth and cell adhesion in the presence of compounds 42-43.

		Planktonic growth			Cell adhesion		
**Cmpd**	**Conc. (μM)**	**A_{600}/min ($x10^{-3}$)**	**Reduction (%)**	**Code (a)**	**N. adhered cells ($x10^{7}$)**	**Reduction (%)**	**Code (b)**
42	0	1.28±0.03[ab]	0.00	0	1.92±0.18[b]	0.00	0
	0.183	1.29±0.01[abc]	0.16	0	3.30±0.53[c]	72.26	1
	1.83	1.31±0.05[abc]	1.96	0	1.84±0.15[b]	-3.76	0
	18.3	1.33±0.01[bc]	3.53	0	1.78±0.18[b]	-7.28	0
	183	1.30±0.01[abc]	1.04	0	1.68±0.29[b]	-12.13	0
	1830	1.03±0.02[d]	-19.39	-1	2.23±0.38[d]	16.50	0
43	0	1.28±0.03[a]	0.00	0	1.92±0.18[bd]	0.00	0
	0.183	1.28±0.04[a]	-0.38	0	2.67±0.45[cd]	39.31	1
	1.83	1.29±0.05[a]	0.55	0	2.00±0.28[bcd]	4.48	0
	18.3	1.30±0.02[a]	1.61	0	1.99±0.33[bd]	4.03	0
	183	1.27±0.03[a]	-1.19	0	1.95±0.34[bcd]	1.86	0
	1830	1.03±0.04[b]	-19.67	-1	1.66±0.33[bd]	-13.31	0

Percentage reduction respect to the negative control is calculated as (ZA-related compound data – control data) x 100 / control data. a) Percentage reduction of the maximum specific growth rate: code (0) x > -10 %; code (-1) -10 % ≤ x < -20 %; code (-2) -20 % ≤ x < -30 %; code (-3) x ≤ -30 %; b) Percentage reduction of the number of adhered cells: code (+1) x > +20 %; code (0) +20 % ≤ x < -20 %; code (-1) -20 % ≤ x < -30 %; code (-2) -30 % ≤ x < -40 %; code (-3) x ≤ -40 %. According to post hoc analysis (Tukey's HSD, $p < 0.05$), data sharing the same letter indicate no significant difference.
