# Supplementary material for: Unravelling the Structural and Molecular Basis Responsible for the Anti-Biofilm Activity of Zosteric Acid
Source: PLoS One. 2015 Jul 1;10(7):e0131519. doi: 10.1371/journal.pone.0131519 (PMC4488431; doi:10.1371/journal.pone.0131519)
Supplement: S2 Table — (PDF) [file pone.0131519.s003.pdf]

S2 Table. Mass spectrometry peptide sequencing.

Accession[a]

P0A8G6

Source[b]	Comp[c]	Peptide confidence[d]	Sequence[e]	Modifications[f]	# PSMs[g]	Xcorr[h]	$[M+H]^+$ (Da)[i]	Charge[j]	m/z (Da)[k]	ΔM (ppm)[l]	RT (min)[m]	# Missed Cleavages[n]
SDS-PAGE	CA	High	TFLDQTGGLWASGALYGK		6	3.66	1884.950	3	628.988	0.62	29.76	0
		High	AVAEGASKVDGAEVVVK		6	3.35	1628.884	3	543.633	-0.98	16.94	1
		High	GGTPYGATTIAGGDGSR		18	3.13	1537.722	2	769.365	-1.37	16.78	0
		High	VLVLYYSmYGHIETmAR	M8(Oxidation); M15(Oxidation)	3	3.13	2078.008	3	693.341	-0.34	24.74	0
		High	TQTAPVATPQELADYDAIIFGTPTR		7	3.11	2676.352	2	1338.679	-0.02	30.55	0
		High	RVPETmPPQLFEK	M6(Oxidation)	9	2.86	1587.820	2	794.414	-0.10	20.87	1
		High	YQGEYVAGLAVK		9	2.83	1297.678	2	649.342	-0.82	21.19	0
		High	VPETmPPQLFEK	M5(Oxidation)	6	2.42	1431.718	2	716.363	-0.33	21.46	0
		High	VDGAEVVVK		8	2.35	915.514	2	458.261	-0.86	15.29	0
		High	FGNmSGQmR	M4(Oxidation); M8(Oxidation)	11	2.32	1059.433	2	530.220	-1.34	6.60	0
		Medium	QPSQEELSIAR		2	1.64	1257.642	2	629.324	-1.40	17.69	0
SDS-PAGE	ZA	High	TQTAPVATPQELADYDAIIFGTPTR		8	5.71	2676.359	2	1338.683	2.81	35.77	0
		High	AVAEGASKVDGAEVVVK		8	5.24	1628.886	2	814.946	0.11	22.44	1
		High	GGTPYGATTIAGGDGSR		10	4.45	1537.724	2	769.366	-0.18	22.45	0
		High	RVPETMPPQLFEK		9	4.19	1571.827	2	786.417	1.09	27.94	1
		High	TFLDQTGGLWASGALYGK		2	4.05	1884.949	2	942.978	0.08	34.59	0
		High	AGGKTQTAPVATPQELADYDAIIFGTPTR		2	3.75	2989.541	3	997.185	4.66	34.49	1
		High	YQGEYVAGLAVK		3	3.47	1297.681	2	649.344	1.91	26.35	0
		High	GGTPYGATTIAGGDGSRQPSQEELSIAR		5	3.35	2776.354	2	1388.680	1.37	26.33	1
		High	RVPETmPPQLFEK	M6(Oxidation)	13	3.27	1587.823	2	794.415	1.90	25.94	1
		High	VPETmPPQLFEK	M5(Oxidation)	3	3.19	1431.721	2	716.364	1.46	26.77	0
		High	VDGAEVVVKR		1	3.09	1071.617	2	536.312	1.27	19.70	1
		High	VDGAEVVVK		5	3.00	915.517	2	458.262	2.60	20.69	0
		High	FGNMSGQMR		3	2.98	1027.446	2	514.227	1.20	21.64	0
		High	VPETMPPQLFEK		8	2.82	1415.725	2	708.366	0.99	29.84	0
		High	FGNMSGQmR	M8(Oxidation)	7	2.80	1043.441	2	522.224	0.91	18.11	0
		High	YQGEYVAGLAVKLNG		1	2.39	1581.829	2	791.418	1.01	29.74	1
		High	QPSQEELSIAR		3	2.21	1257.643	2	629.325	-0.04	22.75	0
		Medium	FGNmSGQmR	M4(Oxidation); M8(Oxidation)	1	1.97	1059.436	2	530.222	1.54	7.38	0
		Medium	LASVFSSTGTGGGQEQTITSTWTTLAHHG MVIVPIGYAAQELFDVSQVR		1	1.70	5148.595	3	1716.870	4.14	43.67	0
2D-PAGE[o]	ZA	High	TQTAPVATPQELADYDAIIFGTPTR		1	5.30	2676.357	2	1338.682	1.99	35.80	0
		High	AVAEGASKVDGAEVVVK		8	5.12	1628.888	2	814.948	1.61	22.45	1
		High	RVPETMPPQLFEK		7	4.12	1571.828	2	786.417	1.64	27.98	1
		High	GGTPYGATTIAGGDGSR		8	3.93	1537.727	2	769.367	1.80	22.16	0
		High	AGGKTQTAPVATPQELADYDAIIFGTPTR		2	3.76	2989.530	3	997.181	0.99	34.50	1
		High	YQGEYVAGLAVKLNG		1	3.61	1581.830	2	791.418	1.55	29.71	1
		High	YQGEYVAGLAVK		3	3.51	1297.681	2	649.344	1.44	26.35	0
		High	VPETmPPQLFEK	M5(Oxidation)	2	3.23	1431.721	2	716.364	1.54	26.78	0
		High	FGNMSGQMR		4	3.05	1027.446	2	514.227	0.96	21.66	0
		High	VDGAEVVVKR		1	2.91	1071.619	2	536.313	3.09	19.73	1
		High	VPETMPPQLFEK		10	2.90	1415.728	2	708.368	2.71	28.81	0
		High	TFLDQTGGLWASGALYGK		2	2.82	1884.957	2	942.982	4.16	34.31	0
		High	VDGAEVVVK		4	2.70	915.517	2	458.262	2.40	20.49	0
		High	RVPETmPPQLFEK	M6(Oxidation)	11	2.64	1587.824	3	529.946	2.47	25.66	1
		High	GGTPYGATTIAGGDGSRQPSQEELSIAR		5	2.54	2776.356	3	926.123	2.06	26.22	1
		High	FGNmSGQmR	M4(Oxidation); M8(Oxidation)	3	2.04	1059.437	2	530.222	2.00	7.29	0
		High	FGNmSGQMR	M4(Oxidation)	5	1.87	1043.443	2	522.225	2.78	17.45	0
		High	QPSQEELSIAR		3	1.86	1257.646	2	629.327	2.19	23.25	0

a) alphanumeric unique protein sequence identifier provided by UniProtKB/Swiss-Prot protein knowledgebase;
b) electrophoresis used to isolate protein;
c) competitor used in the elution step;
d) confidence level associated with the peptide sequence; high confidence is associated to FDR < 0.01, medium confidence to FDR < 0.05;
e) the identified amino acidic peptide sequence;
f) peptides modification at the reported amino acids position;
g) total number of identified peptide sequences (peptide spectrum matches);
h) cross-correlation score for all candidate peptides queried from the database;
i) calculated protonated monoisotopic peptide mass, in Daltons;
j) precursor ion charge state;
k) mass-to-charge ratio of the precursor ion;
l) calculated peptide mass error in parts per million;
m) retention time of the precursor ion, in minutes;
n) number of missed cleavages;
o) spot excised from 2D-PAGE of the fraction collected during competition elution step.
