# Supplementary material for: Correction: Unravelling the Structural and Molecular Basis Responsible for the Anti-Biofilm Activity of Zosteric Acid
Source: PLoS One. 2015 Aug 14;10(8):e0136124. doi: 10.1371/journal.pone.0136124 (PMC4537215; doi:10.1371/journal.pone.0136124)
Supplement: S1 File — (PDF) [file pone.0136124.s001.pdf]

PLOS ONE

RESEARCH ARTICLE

Unravelling the Structural and Molecular Basis Responsible for the Anti-Biofilm Activity of Zosteric Acid

Cristina Cattò[1], Silvia Dell'Orto[2], Federica Villa[1,3], Stefania Villa[2], Arianna Gelain[2], Alberto Vitali[4], Valeria Marzano[5], Sara Baroni[1¤], Fabio Forlani[1]*, Francesca Cappitelli[1]

1 Department of Food Environmental and Nutritional Sciences, Università degli Studi di Milano, Milano, Italy, **2** Department of Pharmaceutical Sciences, Università degli Studi di Milano, Milano, Italy, **3** Center for Biofilm Engineering, Montana State University, Bozeman, MT, United States of America, **4** Institute of Chemistry of Molecular Recognition-UOS Rome, CNR, Roma, Italy, **5** Institute of Biochemistry and Clinical Biochemistry, Catholic University of Rome, Roma, Italy

¤ Current address: Start Up Unit, Department of Experimental Oncology and Molecular Medicine, Fondazione IRCCS Istituto Nazionale dei Tumori, Milano, Italy
* fabio.forlani@unimi.it

CrossMark
click for updates

OPEN ACCESS

Citation: Cattò C, Dell'Orto S, Villa F, Villa S, Gelain A, Vitali A, et al. (2015) Unravelling the Structural and Molecular Basis Responsible for the Anti-Biofilm Activity of Zosteric Acid. PLoS ONE 10(7): e0131519. doi:10.1371/journal.pone.0131519

Editor: Alessio Lodola, University of Parma, ITALY

Received: March 24, 2015

Accepted: June 3, 2015

Published: July 1, 2015

Copyright: © 2015 Cattò et al. This is an open access article distributed under the terms of the Creative Commons Attribution License, which permits unrestricted use, distribution, and reproduction in any medium, provided the original author and source are credited.

Data Availability Statement: All relevant data are within the paper and its Supporting Information files.

Funding: This work was supported by Fondazione Cariplo, grant n. 2011-0277 (FC). The funders had no role in study design, data collection and analysis, decision to publish, or preparation of the manuscript.

Competing Interests: The authors have declared that no competing interests exist.

Abstract

The natural compound zosteric acid, or *p*-(sulfoxy)cinnamic acid (ZA), is proposed as an alternative biocide-free agent suitable for preventive or integrative anti-biofilm approaches. Despite its potential, the lack of information concerning the structural and molecular mechanism of action involved in its anti-biofilm activity has limited efforts to generate more potent anti-biofilm strategies. In this study a 43-member library of small molecules based on ZA scaffold diversity was designed and screened against *Escherichia coli* to understand the structural requirements necessary for biofilm inhibition at sub-lethal concentrations. Considerations concerning the relationship between structure and anti-biofilm activity revealed that i) the *para*-sulfoxy ester group is not needed to exploit the anti-biofilm activity of the molecule, it is the cinnamic acid scaffold that is responsible for anti-biofilm performance; ii) the anti-biofilm activity of ZA derivatives depends on the presence of a carboxylate anion and, consequently, on its hydrogen-donating ability; iii) the conjugated aromatic system is instrumental to the anti-biofilm activities of ZA and its analogues. Using a protein pull-down approach, combined with mass spectrometry, the herein-defined active structure of ZA was matrix-immobilized, and was proved to interact with the *E. coli* NADH:quinone reductase, WrbA, suggesting a possible role of this protein in the biofilm formation process.

Introduction

Direct observation of a wide variety of natural habitats has established that 99% of microorganisms grow in the form of a biofilm, a complex of structured microorganism communities that aggregate on surfaces and that are embedded in a self-produced extracellular polymeric substance [1]. It is reported that biofilms are able to colonize any surface that offers minimal

conditions for life, including all human artefact surfaces such as industrial installations, work benches, water distribution systems, and also medical devices, with devastating consequences in terms of social and economic impacts [1]. In the clinical area, especially, the role of biofilm in the contamination of medical implants is well established; unfortunately, such implants are associated with a high risk of biofilm infection and are major contributors to the morbidity and mortality found among hospitalized patients [2, 3].

The most detrimental property of biofilms is that conventional antimicrobial practices have proven inadequate since, by adopting the sessile mode of life, microorganisms can improve their resistance to antimicrobial agents up to several orders of magnitude [4–6]. In addition, the long term and intensive use of antibiotics and biocides has dramatically supported the development of resistant microbial strains, reducing the possibility of treating biofilm effectively [7]. Increasingly restrictive regulations limiting the use of substances hazardous to human health and the environment have also resulted in several biocides being banned [8–10].

With this perspective, the development of new improved therapeutic solutions able to reduce the incidence of biofilm-associated contamination has become imperative. An innovative approach could be the use of biocide-free anti-biofilm agents with novel targets, unique modes of action and properties that are different from those of currently used antimicrobials [11]. The use of sublethal doses of bio-inspired molecules able to interfere with specific key steps involved in biofilm formation could be an interesting strategy to defeat biofilm formation [12–14]. The use of such strategies might also apply a milder evolutionary pressure in the development of resistance as most virulence traits are not essential for bacterial survival [15].

Zosteric acid or *p*-(sulfoxy) cinnamic acid (ZA), a secondary metabolite produced by the seagrass *Zostera marina*, might be suitable for implementation as a preventive or integrative approach against biofilm formation. At sublethal concentrations, ZA is able to reduce both bacterial and fungal adhesion, and plays a pivotal role in shaping biofilm architecture. This can be seen through the reduction of biofilm biomass and thickness, and the thwarting of budded-to-hyphal-form transition. Additionally, ZA extends the performance of antimicrobial agents, and shows cytocompatibility towards soft and hard tissue [16–18]. A comparative proteomic study on *E. coli* cells exposed to ZA has shown that, in bacterial cells, ZA acts as an environmental cue to global stress, promoting the expression of various protective proteins, the production of the signal molecule autoinducer-2, and the synthesis of flagella to escape from adverse conditions [19].

Despite such promising performance there are currently two main bottlenecks limiting the exploitation of ZA as a preventive strategy to counteract the formation of biofilm on abiotic surfaces: i) the structural elements required for anti-biofilm activity remain unclear and ii) the cellular targets for ZA are still unexplored.

In this follow-up study, a 43-member library of small molecules based on ZA-scaffold diversity was designed and screened against *E. coli*, used as the model bacterial biofilm system. Thus, the aim of this study was to identify important structural determinants for the ZA anti-biofilm activity, and to eventually develop ZA analogues with improved anti-biofilm activity. In order to functionally exploit the information arising from the molecule library analysis, the resulting active structure of the ZA-scaffold was matrix-immobilized and its ability to interact with a cell component was evaluated. On the assumption that the primary events triggering the processes related to the anti-biofilm activity of ZA rely on ZA's interaction with a protein, a protein pull-down approach was developed. The identification of the cellular target for ZA is thus a crucial step to an understanding of the mode of action, and for the structure-led design and improvement of any potential therapeutic compound and material. Our work provides not only the first identification of the cellular target for the active structure of ZA, but also

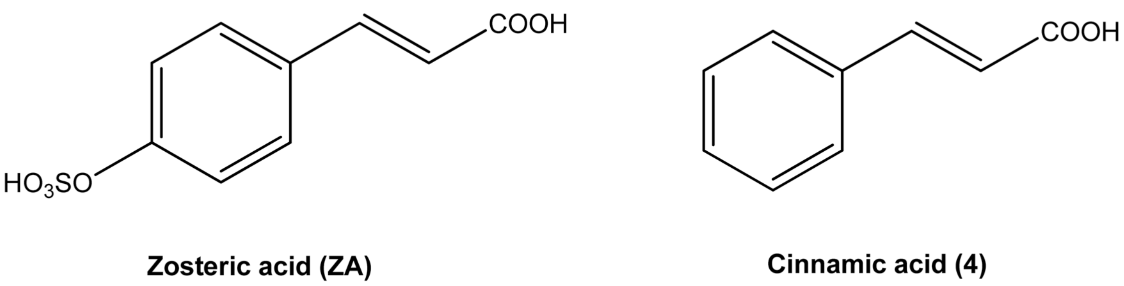

Fig 1. Structural formula of zosteric acid (ZA) and cinnamic acid (4).

doi:10.1371/journal.pone.0131519.g001

convincing evidence that its mode of action is likely to result from a synergistic effect arising from a perturbation of oxidative homeostasis.

Materials and Methods

Zosteric acid related analogues

The compounds used in this study are listed in Figs 1 and 2. Some compounds were purchased from Sigma-Aldrich, others were prepared following slightly modified literature procedures (S1 Protocol). All the reagents, including solvents, were purchased from Sigma-Aldrich, and were used without any further purification. Reactions involving air-sensitive reagents were performed under nitrogen atmosphere and anhydrous solvents were used when necessary. The Biotage-Initiator microwave synthesizer was used. Reactions were monitored by Thin Layer Chromatography (TLC) analysis on aluminium-backed Silica Gel 60 plates (0.2 mm, Merck), and were visualized under a UV lamp operating at wavelengths of 254 and 365 nm. Visualization was aided by opportune staining reagents. Intermediates and final compounds were purified by flash chromatography using Merck Silica Gel 60 (70–230 mesh). The purity of the final compounds was determined by High Performance Liquid Chromatography (HPLC) analysis and

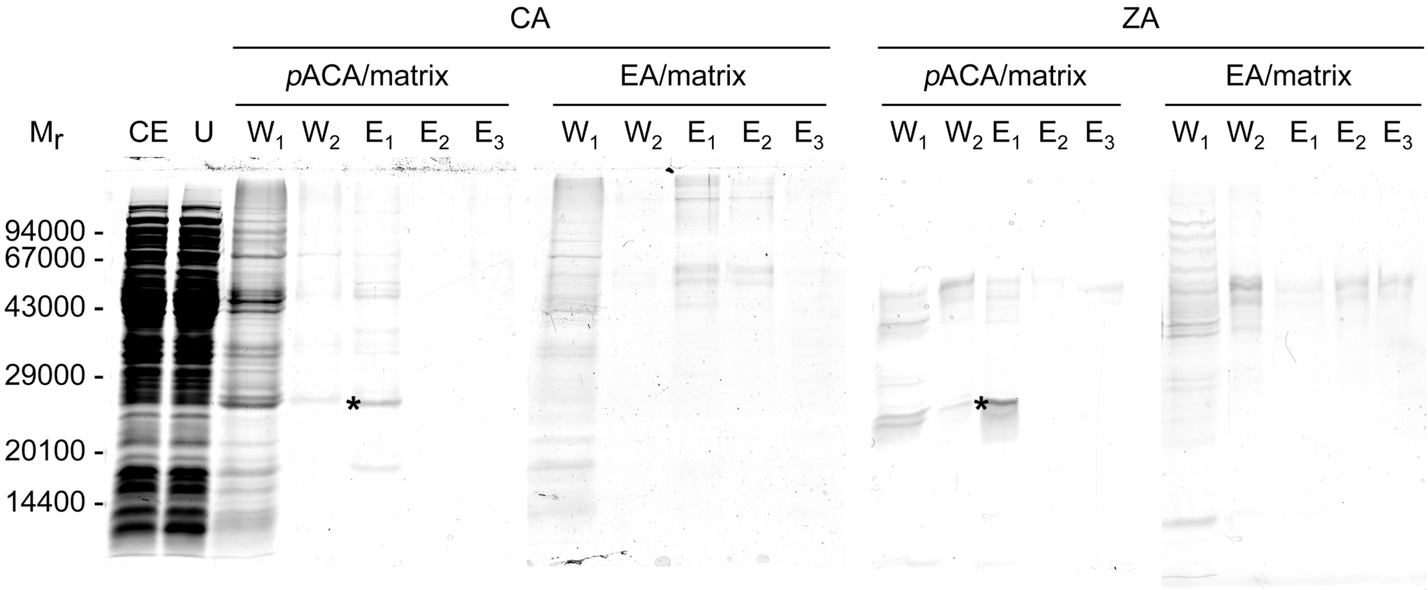

Fig 2. Structures of cinnamic acid analogues used in the present study.

doi:10.1371/journal.pone.0131519.g002

was > 95%. ^{1}H and ^{13}C Nuclear Magnetic Resonance (NMR) spectra were recorded at room temperature on a Varian 300 MHz Oxford instrument. $CDCl_3$, CD_3OD, D_2O, acetone-d6 and DMSO-d6 were used as deuterated solvents for all spectra runs. Chemical shifts are expressed in ppm from tetramethylsilane resonance in the indicated solvent (TMS: 0.0 ppm), and coupling constants (J-values) are given in Hertz (Hz). ^{1}H NMR data are reported in the following order: ppm, multiplicity (s, singlet; d, doublet; t, triplet; q, quartet; m, multiplet; br, broad), and number of protons. Melting points and NMR data are consistent with literature data.

Synthesis of cinnamic acid derivatives

Zosteric acid was synthesized as already described in a previous work [16]. The *cis* isomer **38** was obtained starting from *cis* 4-hydroxycinnamic acid **32** under microwave irradiation in the presence of sulfur trioxide pyridine complex in acetonitrile (Fig 3). The final product was isolated as sodium salt. Most of the substituted cinnamic acid derivatives were prepared in high yield (> 90%) by the Knoevenagel-Doebner procedure. In detail, compounds **2, 7, 8**, **11** and **17** were obtained through a one-pot reaction between the suitable substituted benzaldehyde and malonic acid in refluxing pyridine to induce decarboxylation (Fig 4) [20]. The *trans* geometries of the ethenyl π-bonds were confirmed by proton-proton coupling constants. *Cis* cinnamic acid **37** was synthesized from the commercially available ethyl phenylpropiolate. The subsequent hydrogenation of alkyne in the presence of the Lindlar catalyst and pyridine in methanol led to the corresponding *cis*-alkene **36**. Then the ester group was hydrolyzed under alkaline conditions to provide the final compound **37** (Fig 5). Esters of cinnamic acid in *cis* (**33**) or in *trans* configuration (**23**, **24**) were prepared by Fischer esterification of the carboxylic group [21]. The protection of the hydroxyl group as methyl ether in the presence of iodomethane in dry *N*,*N*-dimethylformamide provided the compounds **35**. The hydrolysis of the ester was performed in alkaline conditions to obtain compound **34** (Fig 3).

Escherichia coli strain and growth condition

The well characterized *Escherichia coli* K-12 wild-type strain (ATCC 25404) was used as a model system for the bacterial biofilms. The strain was stored at –80°C in suspensions containing 20% glycerol and 2% peptone, and was routinely grown in Luria-Bertani broth (LB, Sigma-Aldrich) at 30°C for 16 h.

Planktonic growth in the presence of ZA-related compounds

Planktonic growth of *E. coli* in LB medium supplemented with 0 (negative control), 0.183, 1.83, 18.3, 183 and 1830 μM of the synthesized molecules and 3% of DMSO were carried out in 384-well microtiter plates. Growth curves at 30°C were generated using the PowerWave XS2 microplate reader (Biotek). Growth was followed by measuring the absorbance at 600 nm (A_{600}) every 10 min for over 24 h in wells inoculated with 3 μl (3% vol/vol) of an overnight culture (final concentration 10^7 cells/mL). An absorbance-based growth kinetics was constructed by plotting the A_{600} of suspensions minus the A_{600} of the non-inoculated medium against incubation time. The polynomial Gompertz model [22] was used to fit the growth curves to calculate the maximum specific growth rate (A_{600}/min), using GraphPad Prism software (version 5.0, San Diego, CA, USA). Six biological replicates of each treatment were performed. The obtained data were normalized to the negative control and reported as the mean of these. The percentage reduction, compared to the control, was also calculated. Molecules able to reduce the maximum specific growth rate of *E. coli* planktonic cells with respect to the negative control were coded as follows: less than 10% as 0, between 10% and 20% as—1, between 20% and 30% as—2, and more than 30% as—3.

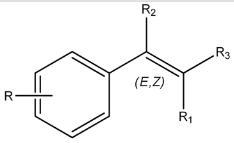

1 - 42

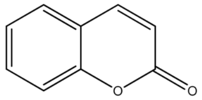

43

Code	Cmpd	R	R_1	R_2	R_3	*E/Z*
**ZA**	Zosteric acid	4-OSO_3H	H	H	COOH	*E*
**1**	Coumaric acid *	4-OH	H	H	COOH	*E*
**2**	4-Methylcinnamic acid	4-CH_3	H	H	COOH	*E*
**3**	4-Formylcinnamic acid *	4-COH	H	H	COOH	*E*
**4**	Cinnamic acid *	-	H	H	COOH	*E*
**5**	4-Methoxycinnamic acid *	4-OCH_3	H	H	COOH	*E*
**6**	4-Fluorocinnamic acid *	4-F	H	H	COOH	*E*
**7**	4-Cyanocinnamic acid	4-CN	H	H	COOH	*E*
**8**	4-Carboxycinnamic acid	4-COOH	H	H	COOH	*E*
**9**	4-Chlorocinnamic acid *	4-Cl	H	H	COOH	*E*
**10**	4-Aminocinnamic acid *	4-NH_2	H	H	COOH	*E*
**11**	4-(Trifluoromethyl)cinnamic acid	4-CF_3	H	H	COOH	*E*
**12**	4-Nitrocinnamic acid *	4-NO_2	H	H	COOH	*E*
**13**	3-Chlorocinnamic acid *	3-Cl	H	H	COOH	*E*
**14**	3-Hydroxycinnamic acid *	3-OH	H	H	COOH	*E*
**15**	3,4-Dichlorocinnamic acid *	3-Cl; 4-Cl	H	H	COOH	*E*
**16**	2,4-Dichlorocinnamic acid *	2-Cl; 4-Cl	H	H	COOH	*E*
**17**	2-Chlorocinnamic acid	2-Cl	H	H	COOH	*E*
**18**	2-Methoxycinnamic acid *	2-OCH_3	H	H	COOH	*E*
**19**	2-Chloro-4-fluorocinnamic acid *	4-F; 2-Cl	H	H	COOH	*E*
**20**	Caffeic acid *	3-OH; 4-OH	H	H	COOH	*E*
**21**	2-Hydroxycinnamic acid *	2-OH	H	H	COOH	*E*
**22**	3-Hydroxy-4-aminocinnamic acid *	4-NH_2; 3-OH	H	H	COOH	*E*
**23**	Methyl coumarate	4-OH	H	H	$COOCH_3$	*E*
**24**	Methyl 4-methoxycinnamate	4-OCH_3	H	H	$COOCH_3$	*E*
**25**	Ethyl 2-hydroxycinnamate *	2-OH	H	H	$COOCH_2CH_3$	*E*
**26**	Methyl cinnamate *	-	H	H	$COOCH_3$	*E*
**27**	Ethyl β-methylcinnamate *	-	CH_3	H	$COOCH_2CH_3$	*E*
**28**	Ethyl cinnamate *	-	H	H	$COOCH_2CH_3$	*E*
**29**	Cinnamaldehyde *	-	H	H	CHO	*E*
**30**	α-Methyl cinnamaldehyde *	-	H	CH_3	CHO	*E*
**31**	Cinnamyl alcohol *	-	H	H	CH_2OH	*E*
**32**	*Cis* coumaric acid *	4-OH	H	H	COOH	*Z*
**33**	*Cis* methyl coumarate	4-OH	H	H	$COOCH_3$	*Z*
**34**	*Cis* 4-methoxycinnamic acid	4-OCH_3	H	H	COOH	*Z*
**35**	*Cis* methyl 4-methoxycinnamic acid	4-OCH_3	H	H	$COOCH_3$	*Z*
**36**	*Cis* ethyl cinnamate	-	H	H	$COOCH_2CH_3$	*Z*
**37**	*Cis* cinnamic acid	-	H	H	COOH	*Z*
**38**	*Cis* zosteric acid	4-OSO_3H	H	H	COOH	*Z*
**39**	3-(4-Methoxy)phenylpropionic acid *	4-OCH_3	H	H	COOH	-
**40**	3-Phenylpropionic acid *	-	H	H	COOH	-
**41**	3-(3,4-Dihydroxy)phenylpropionic acid *	3-OH; 4-OH	H	H	COOH	-
**42**	3-(4-Hydroxy)phenylpropionic acid *	4-OH	H	H	COOH	-
**43**	Coumarin *	-	H	H	-	-

* commercially available compounds

Fig 3. Synthetic scheme of compounds 23, 24, 33, 34, 35, 38. Reagents and conditions: i) from **1** for **23**, from **5** for **24**, from **32** for **33**: H_2SO_4, MeOH, reflux, 1 h; ii) from **32**: Py · SO_3, DMF dry, 120°C, 25 min; iii) CH_3I, anhydrous K_2CO_3, dry DMF, reflux, 1.5 h; iv) 1N NaOH, EtOH.

doi:10.1371/journal.pone.0131519.g003

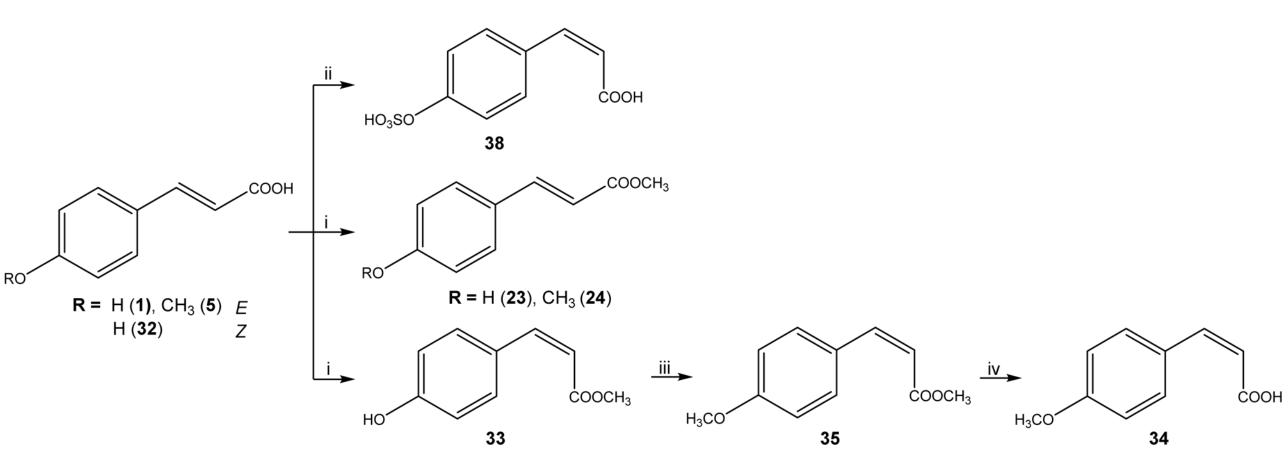

Fig 4. Synthetic scheme of compounds 2, 7, 8, 11, 17. Reagents and conditions: i) malonic acid, Py, piperidine, reflux, 80–180 min.

doi:10.1371/journal.pone.0131519.g004

The ability of bacteria to grow with each compound as the sole carbon and energy source was also tested using a mineral medium (KH_2PO_4 30 g/L, Na_2HPO_4 70 g/L, NH_4Cl 10 g/L, pH 7) supplemented with 1830 μM of each synthesized molecule and 3% of DMSO. Bacteria were added to a final concentration of 10^7 cells/mL and grown at 30°C for 72 h. The positive control was the mineral medium supplemented with glucose at both 1830 μM and 10000 μM concentration. Microbial growth was followed by measuring A_{600}. All the experiments were repeated three times.

Biofilm assay in the presence of ZA-related compounds

The effects of ZA-related compounds on cell adhesion were assessed quantitatively using fluorochrome-labeled cells in hydrophobic 96-well black sided plates as previously reported by Villa *et al.* [16]. Briefly, 200 μL of phosphate buffered saline (PBS, 0.01 M phosphate buffer, 0.0027 M potassium chloride pH 7.4, Sigma-Aldrich) containing 10^7 cells supplemented with 0 (negative control), 0.183, 1.83, 18.3, 183 and 1830 μM of each synthesized molecule and 3% of DMSO were placed in microtiter plate wells. Cells were incubated 18 h at 30°C. The microtiter plate wells were washed twice with 200 μL PBS, and adhered cells were stained using 10 μg/mL 4,6-diamidino-2-phenylindole (Sigma-Aldrich) in PBS for 20 min in the dark at room temperature. Fluorescence intensity was measured using the fluorometer VICTOR X Multilabel Plate

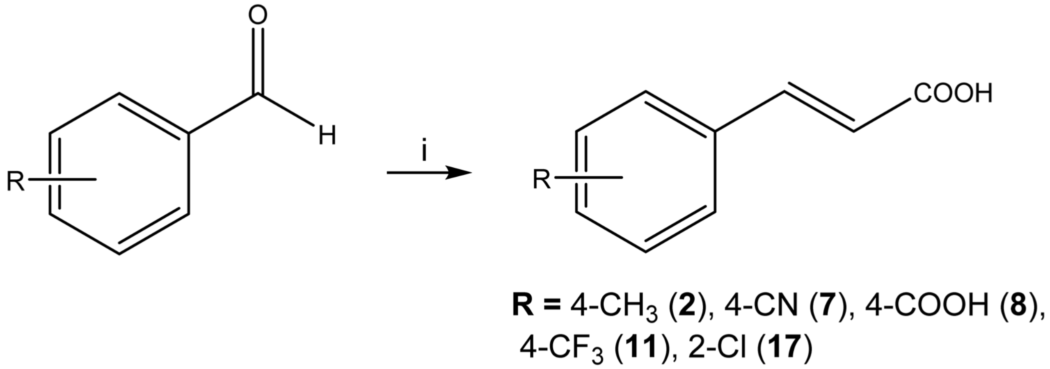

Fig 5. Synthetic scheme of compounds 36, 37. Reagents and conditions: i) Lindlar catalyst, H_2, pyridine, MeOH, r.t., 16 h; ii) 1N NaOH, EtOH/THF (1:1), r. t., 12 h.

doi:10.1371/journal.pone.0131519.g005

Readers (Perkin Elmer) at excitation wavelength of 335 nm and emission wavelength of 433 nm. A standard curve of fluorescence intensity versus cell number was determined and used to quantify the anti-biofilm performance of the ZA-related compounds. Six biological replicates of each condition were performed. The experiment was repeated four times. Obtained data were normalized to the negative control and reported as the mean of these data. Percentage reduction in comparison to the control was also calculated. Molecules able to reduce the number of *E. coli* adhered cells by less than 20% with respect to the negative control were coded 0, between 20% and 30% as—1, between 30% and 40% as—2 and more than 40% as—3. Molecules able to increase the number of adhered cells by more than 20% with respect to the negative control were coded + 1.

Statistical analysis

Analysis of variance (ANOVA) via a software run in MATLAB (Version 7.0, The MathWorks Inc., Natick, USA) was applied to statistically evaluate any significant differences among the samples. The ANOVA analysis was carried out after verifying whether the data satisfied the assumptions of i) independence, ii) normal distribution and iii) homogeneity of variances. Tukey's honestly significant different test (HSD) was used for pairwise comparison to determine the significance of the data. Differences were considered significant for $p < 0.05$.

E. coli protein extraction

An *E. coli* culture was prepared as reported in the section '*Escherichia coli* strain and growth condition'. The *E. coli* culture was centrifuged at 5000 *g* for 15 min at 4°C and collected cells (~ 2.5 g, wet weight) were washed 3 times with PBS and resuspended in 200 mM Tris-HCl, 40 mM NaCl, pH 7.5 (protein extraction buffer) to a final concentration of 250 mg cells/mL. Soluble protein extract was obtained by sonication (seven 1-min sonication cycles at 22 μm amplitude followed by 2-min cooling periods, all on ice-bath, in Soniprep 150) and cell debris was removed by centrifugation at 10000 *g* for 45 min at 4°C.

Matrix immobilization of *p*-amino cinnamic acid

Five milliliters of drained NHS-Activated Sepharose 4 Fast Flow (GE Healthcare Life Sciences) were washed 10 times with 15 mL of cold 1 mM HCl and then suspended in 2.5 mL of 0.2 M $NaHCO_3$, 0.5 M NaCl, 3% DMSO, pH 8.3 (coupling buffer), containing 0.4 M *p*-amino cinnamic acid (*p*-ACA). The suspension was mixed in a rotary shaker overnight at 4°C. After removal of the coupling mixture, the remaining unreacted groups were blocked with 0.5 M ethanolamine, 0.5 M NaCl, pH 8.3 (5 mL) and 10 subsequent washing steps were performed according to the manufacturer's instructions. The matrix (*p*-ACA/matrix) was stored in 20% ethanol (3 mL) at 4°C. To generate the control matrix (EA/matrix), *p*-ACA was omitted in the coupling mixture.

Fluorescence analysis

Fluorescence measurements were carried out in a Perkin-Elmer LS 50B spectrofluorometer equipped with PTP-1 Fluorescence Peltier System. Excitation and emission spectra (250–600 nm) were recorded at 50 nm/min, with both emission and excitation slit widths set at 3.0 nm, and at 20°C. Fluorescence spectra of *p*-ACA free in solution were recorded by dissolving different concentrations (0.2–5.0 mM) of *p*-ACA in 0.4 M $NaHCO_3$, 1 M NaCl (pH 8.3). Fluorescence spectra of *p*-ACA/matrix and EA/matrix suspensions were recorded under continuous magnetic stirring.

Matrix hydrolysis and analysis of hydrolysates

One mL (drained volume) of the matrix samples was hydrolyzed by treatment with 4 M NaOH aqueous solution (1 mL). The suspensions were stirred 4 h at room temperature. Reactions were monitored by thin layer chromatography (TLC) analysis on aluminium-backed Silica Gel 60 plates (0.2 mm, Merck) and were visualized under UV lamp (λ = 254 nm). Visualization was aided by opportune staining reagents. The matrix was filtered off and washed. The aqueous layer was acidified to pH 1 with 2 M HCl and then extracted with ethyl acetate (3 x 1 mL). The combined organic layers were dried over anhydrous sodium sulfate, residual solvent was evaporated under reduced pressure and submitted to mass spectrometry analysis.

Matrix hydrolysates were resuspended in 100% methanol, further diluted with 0.1% formic acid in water and analysed by liquid chromatography-electrospray ionization-mass spectrometry (LC-ESI-MS) on an Ultimate 3000 Micro HPLC apparatus (Dionex, Sunnyvale, CA, USA) equipped with a FLM-3000-Flow manager module directly coupled to a LTQ Orbitrap XL hybrid FT mass spectrometer (Thermo Fisher Scientific, Waltham, MA, USA). Reverse-phase chromatography was performed on a Jupiter C18, 5 μm, 150 x 1.0 mm column (Phenomenex, Torrance, CA, USA) and a 31 min run (gradient 0 to 40% acetonitrile in water with 0.1% formic acid over 20 min) at a flow rate of 80 μL/min. Mass spectra were collected at 60000 resolution in the Orbitrap analyser (mass range 50–1000 *m/z*) in positive ion mode. High resolution MS data were elaborated manually using the Xcalibur Qual Browser software (version 2.2, Thermo Fisher Scientific).

Protein pull-down

Functionalized matrix (5 mL, drained volume) was washed once with 5 mL water and then with 5 mL of protein extraction buffer. To ease the removal of the liquid phase by matrix decanting, centrifugation at 700 *g* for 2 min at room temperature was applied after each washing step. The washed matrix was incubated with freshly-prepared soluble protein extract (~ 9.6 mL; ~ 90 mg protein) for 2.5 h at room temperature in a rotary shaker. The incubation mixture was then packed into a 15 mL chromatography column (i. d. 10 mm) and washed with protein extraction buffer until absorbance at 280 nm was null (~ 100 mL), at a flow rate of 1.5 mL/min to remove unbound proteins. Proteins specifically bound to the functionalized matrix were recovered by competitive elution with 60 mM competitor (15 mL; sodium cinnamate or sodium zosterate) in protein extraction buffer, followed by 50 mL of 200 mM Tris-HCl, 1 M NaCl, pH 7.5. A compact single beam UV-detector (Uvicord SII, 280 nm filter; GE Healthcare) was employed for flow-through monitoring and fractions were collected during the course of the chromatography. To increase the protein concentration, the fraction aliquots were precipitated using cold 13% trichloroacetic acid (TCA), centrifuged at 13000 *g* for 30 min at 4°C and subsequently washed with cold acetone.

Other procedures

Protein concentration was determined using the Bradford method, using bovine serum albumin as standard [23]. Electrophoretic analysis (SDS-PAGE) was performed under denaturing and reducing conditions according to Laemmli [24]. TCA-precipitated proteins were dissolved in Laemmli sample buffer and submitted to thermal denaturation before SDS-PAGE. Coomassie-stained bands were manually excised from gels and subjected to mass spectrometry analysis.

Mass spectrometry analysis and protein identification

Excised bands and spots were subjected to trypsin digestion as previously described [25] and the trypsin digested peptides were analyzed by liquid chromatography-electrospray ionization-tandem mass spectrometry (LC-ESI-MS/MS) using the same HPLC and LTQ Orbitrap mass spectrometer previously reported in "Matrix hydrolysis and analysis of hydrolysates". Reverse-phase chromatography was performed on the same column and at the same flow rate but under different chromatographic conditions: linear gradient 1.6 to 44% acetonitrile in water with 0.1% formic acid over 40 min and total LC-run of 61 min. Mass spectra were collected in positive ion and data dependent scan mode (MS scan at 60000 of resolution in the Orbitrap, mass range 300–2000 *m/z*, and MS/MS scan on the three most intense peaks in the linear ion trap). Selected peptide charge states were isolated with a width of *m/z* 2, and were activated for 30 msec using 35% normalized collision energy and an activation q of 0.25. Protein identification was achieved using the embedded ion accounting algorithm (Sequest HT) of the software Proteome Discoverer (version 1.4, Thermo Fisher Scientific) after searching a UniProtKB/Swiss-Prot Protein Knowledgebase [release 2013_12 of 11-Dec-13; taxonomical restriction: *Escherichia coli* (strain K12), 4431 sequence entries]. The search parameters were 10 ppm tolerance for precursor ions and 0.6 Da for product ions, 1 missed cleavage, carbamydomethylation of cysteine as fixed modification, the oxidation of methionine as variable modification and on a decoy database search calculated false discovery rate under 5%.

Results and Discussion

Chemical perspective of ZA mode of action

The main goal of this study was to identify structural determinants for the anti-biofilm activity of ZA, and highlight features that could be useful in the design of new derivatives endowed with better anti-biofilm activity than ZA.

Therefore, a 43-member library of small molecules based on the ZA-scaffold was designed and screened against *Escherichia coli*. Compounds were characterized by introduction of substituents at different positions on the phenyl ring and making several side-chain modifications, such as removal of unsaturation and substitution of the carboxylic acid with an alcohol, an aldehyde and ester functionalities. Moreover, both E/Z isomers were prepared in order to explore the role of the double bond. Of all the concentrations tested (0.183, 1.83, 18.3, 183, 1830 μM), the concentration at which ZA performed its best anti-biofilm activity was 1830 μM [16].

Before evaluating the anti-biofilm activity of ZA derivatives, we investigated both their ability to act as a carbon and energy source and their potential impact on the planktonic growth of the model bacterium *E. coli*. To dissolve the molecules in the aqueous medium it was necessary to use DMSO, so its possible impact on *E. coli* planktonic growth and cell adhesion was assessed. It was found that 3% DMSO successfully dissolved the molecules in the media, and had no effect on either planktonic growth or cell adhesion.

In the first steps of the study the ability of *E. coli* to grow with 43 ZA-related compounds at 1830 μM as the sole carbon and energy source was tested and compared with a positive control using glucose at the same concentration. The *E. coli* in the positive control did not grow, and showed an A_{600} lower than 0.05 after 48 h incubation. Indeed, the minimum glucose concentration in a mineral medium that supported *E. coli* growth was 10 mM (A_{600} = 0.201 ± 0.007). Consequently, *E. coli* with glucose 10 mM was taken to be the positive control. *E. coli* did not grow in the presence of each of the ZA-related compounds as the sole carbon and energy source, and showed an A_{600} lower than 0.05 after 48 h incubation with each molecule.

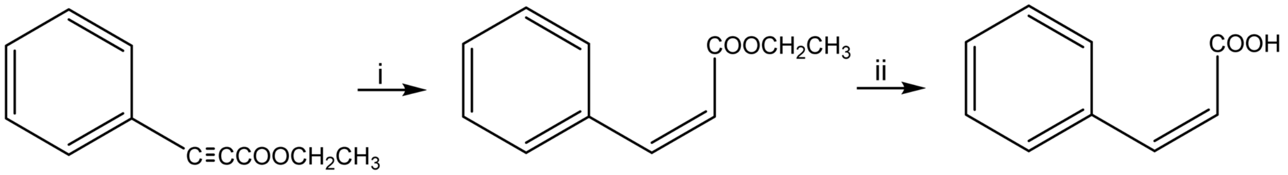

36 **37**

Fig 6. Biological activity of ZA-related compounds. Biological activity of each ZA-related compound at the different tested concentrations. For an understanding of the meaning of each trend refer to the 'Results and discussion' section. Yellow: no biological activity; Green: anti-biofilm activity; Blu: promotion of biofilm formation; Red: biocidal effect.

doi:10.1371/journal.pone.0131519.g006

The ability of the 43 ZA-related compounds to affect *E. coli* planktonic growth was also evaluated. The *E. coli* maximum specific growth rates in the presence of each ZA-related compound at 0.183, 1.83, 18.3, 183 and 1830 μM were calculated and compared to a negative control grown without the molecule, using the ANOVA statistic test (S1 Table).

On the basis of these results, all the derivatives were evaluated for their ability to affect surface cell adhesion. Thus the number of adhered cells in the presence of each ZA-related compound, at 0.183, 1.83, 18.3, 183 and 1830 μM was assessed quantitatively using fluorochrome-labelled cells, and compared to a negative control in the absence of the molecule, using the ANOVA statistic test (S1 Table).

With the aim of identifying trends, planktonic growth and cell adhesion data were grouped to obtain a global picture of the biological activity of each ZA-related compound at the different concentrations (Fig 6). The compounds showed: i) no biological activity (no reduction of the number of adhered cells and no planktonic growth effect), ii) anti-biofilm activity (reduction of the number of adhered cells and no planktonic growth effect), iii) increased biofilm formation (increase in the number of adhered cells and no planktonic growth effect), and iv) biocidal effect (in contrast to the above trends, planktonic growth was inhibited while the number of adhered cells either increased or decreased). Five molecules (**7**, **12**, **18**, **19**, **39**) induced no biological activity at low and middle concentrations, or biocidal effect at the maximum concentration (Trend 1). Nine molecules (**24**, **25**, **27**, **29–31**, **40**, **42**, **43**) promoted biofilm formation at the lowest concentration but showed no biological activity or biocidal effect at middle and high concentrations (Trend 2). Nineteen molecules (**1–6**, **8–11**, **13–15**, **20**, **21**, **23**, **26**, **28**, **41**) had no biological activity at low concentration, showed an anti-biofilm activity at middle concentrations and induced biocidal effect at the maximum concentration (Trend 3). Eight molecules (**ZA**, **16**, **17**, **22**, **33–36**) promoted biofilm formation at the lowest concentration, showed an anti-biofilm activity at middle concentrations, and induced a biocidal effect at the maximum concentration (Trend 4). Finally, molecules **32**, **37** and **38** showed a peculiar trend (Trend 5): molecule **32** showed anti-biofilm activity at the lowest concentration, no biological activity at middle concentrations and biocidal effect at the highest concentration, molecule **37** promoted biofilm formation at concentrations 0.183, 1.83 and 183 μM, inhibited biofilm formation at 18.3 μM and induced a biocidal effect at the highest concentration; molecule **38** promoted biofilm formation at all tested concentrations except for the 183 μM, where there was no biological activity.

For each ZA-related compound a global anti-biofilm performance value was also calculated as (sum of cell adhesion codes of all concentrations) - (sum of planktonic growth codes of all concentrations) (Fig 7). This value takes into account i) the occurrence that the ZA-related compound induced a different bacterial response depending on the concentration, ii) the positive contribution of concentrations that reduce the number of adhered cells; iii) the negative

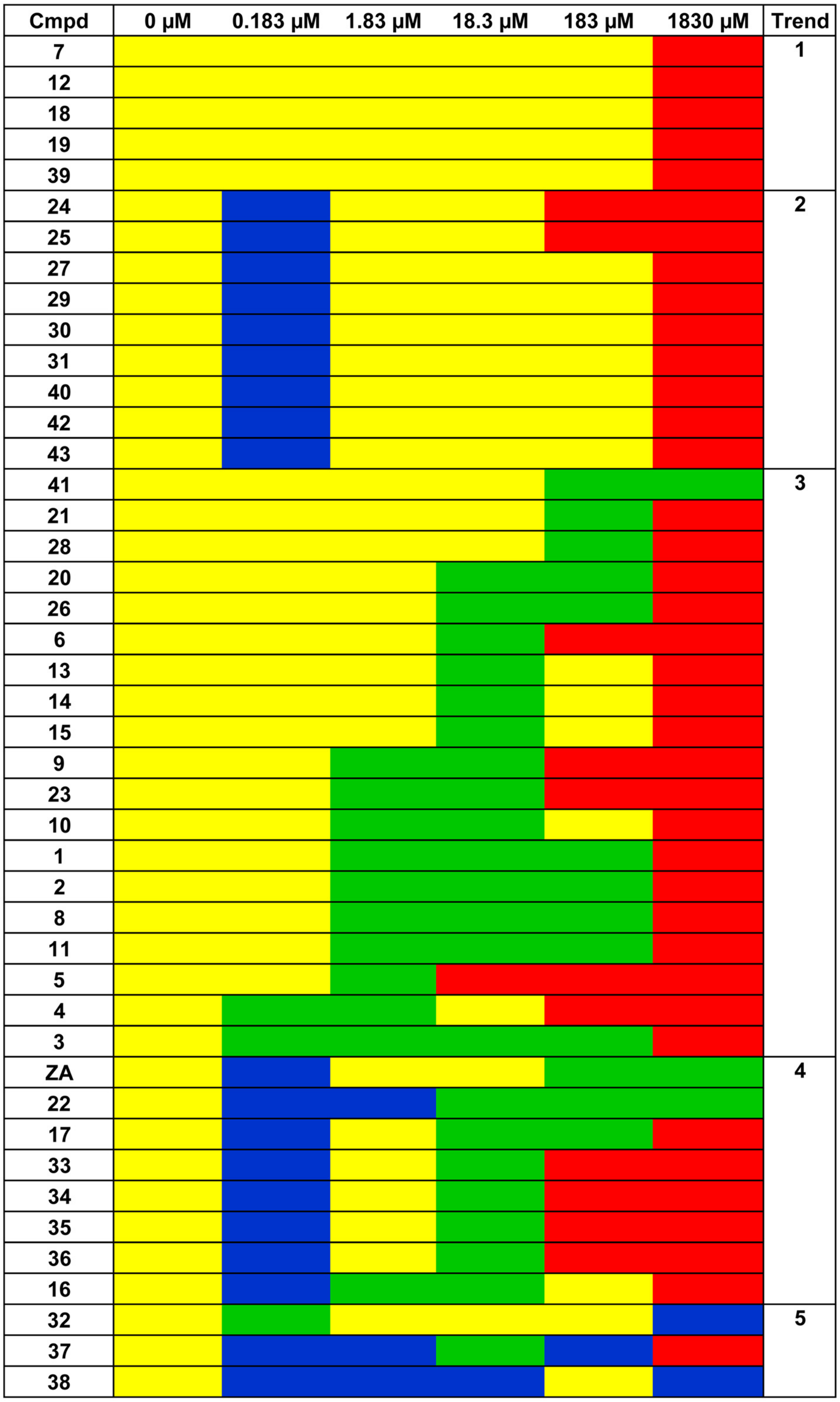

Fig 7. Global anti-biofilm performance of ZA-related compounds and structures with the most significant anti-biofilm performance. Global anti-biofilm performance value of each ZA-related compound calculated as (sum of cell adhesion codes of all concentrations)-(sum of planktonic growth codes of all concentrations). Values equal to 0 were considered without anti-biofilm performance, below 0 were considered globally able to exert an anti-biofilm activity, and above 0 were considered able to improve biofilm

performance. Yellow: no anti-biofilm performance; Light orange: little anti-biofilm performance; Medium orange: moderate anti-biofilm performace; Dark orange: optimal anti-biofilm performance; Light green: little improvement of anti-biofilm performance; Medium green: moderate improvement of anti-biofilm performance; Dark green: optimal improvement of anti-biofilm performance.

doi:10.1371/journal.pone.0131519.g007

contribution of concentrations that enhances the number of adhered cells; iv) the negative contribution of concentrations that reduces planktonic growth (the inhibition of planktonic growth negatively affected the anti-biofilm performance). Molecules with an anti-biofilm performance value i) equal to 0 were considered globally without anti-biofilm performance; ii) below 0 were considered globally able to exert an anti-biofilm activity (= - 1: little anti-biofilm performance; = - 2: moderate anti-biofilm performance; $\leq$ - 3: optimal anti-biofilm performance), iii) above 0 were considered globally able to improve biofilm performance (= +1: little improvement of biofilm performance; = +2: moderate improvement of biofilm performance; $\geq$+3: optimal improvement of biofilm performance). Four molecules (**7, 12, 14, 32**) presented anti-biofilm performance equal to 0. Twenty molecules presented anti-biofilm performance below 0 (= - 1: **16**; = - 2: **5**, **6**, **13**, **21**, **23**, **28**; $\leq$ - 3: **ZA**, **1–4**, **8–11**, **17**, **20**, **22**, **41**). Twenty molecules presented anti-biofilm performance above 0 (= + 1: **19**, **26**, **31**, **33**, **36**; = + 2: **15**, **25**, **27**, **39**, **42**, **43**; $\geq$ + 3: **18**, **24**, **29**, **30**, **34**, **35**, **37**, **38**, **40**).

The molecules grouped on the basis of both their biological activity and anti-biofilm performance value allowed the recognition of some chemical structure elements required to exert anti-biofilm activity.

Interestingly, as reported in the S1A Table, cinnamic acid **4**, a natural compound obtained by cinnamon lacking the sulfate monoester group, showed interesting anti-biofilm activity at a 1000-fold lower inhibitory concentration than ZA (0.183 *vs* 183 μM).

The substitution pattern of the phenyl ring and the nature of the substituents were found to have a significant effect on the biofilm inhibitory activity. In the first instance, a broad variety of substituents with different electronic and steric properties were introduced at position 4 on the phenyl ring. Compounds (**1–3**, **5**, **6**, **8–11**) showed interesting activity (Trend 3) compared to the unsubstituted cinnamic acid **4**, meanwhile the presence of cyano (**7**) and nitro (**12**) groups caused a loss in activity (Trend 1).

Comparing the derivatives bearing a hydroxyl group (**1**, **14**), as well as the chlorine atom (**9**, **13**), respectively in positions 4 and 3 on the phenyl ring, highlighted that the substitution at the *para* position was preferred (**1** and **9** more active than **14** and **13**), although the same trend was still present. On the contrary, the substitution at position 2 was not well tolerated. Indeed, as for compound **17** the activity was reduced 10-fold compared to **9**, while in the other cases the activity was almost completely abolished. To determine whether the introduction of a second substituent on the phenyl ring would enhance activity, a series of disubstituted derivatives (**15**, **16**, **19**, **20**, **22**) were synthesized. In general, each disubstituted compound was found to have different behavior and lower anti-biofilm activity compared to the corresponding monosubstituted compound. This effect was most evident for the 3,4-dichlorocinnamic acid **15**, whose activity was 10-fold lower than the monosubstituted counterpart **9**.

Following this, the side chain modifications were also explored. In general, the unsubstituted compounds having the carboxylic function protected as esters (**26**, **28**) showed lower activity in comparison to cinnamic acid **4**, and particularly the ethyl ester **28** was less active than methyl ester **26**. Besides, among the *para*-substituted cinnamic acid esters (**23–24**), the presence of a hydroxyl group (**23**), seemed to be important for the anti-biofilm activity as the corresponding 4-methoxy derivative **24** was inactive. Moreover, derivatives bearing an aldehyde (**29**, **30**) or an alcohol (**31**) to replace the carboxylic acid functionality were inactive

(Trend 2), confirming that the carboxylic moiety was essential for good anti-biofilm activity. To investigate the importance of the configuration of the double bond, the majority of *cis* derivatives (**32–38**) were synthesized and found to have different behavior (Trends 4, 5) compared to the *trans* counterparts. Interestingly, exceptions to this rule were the *cis* isomer of ZA **38** and cinnamic acid **37**, which had lost their anti-biofilm activity (Trend 5). Additionally, specific concentrations of **37** and **38** seemed to enhance the biofilm formation.

Similarly, some saturated derivatives (**39–42**) were chosen to investigate the role of the double bond. All the compounds exhibited the same trend (Trend 2) except for compound **41**, a catechol derivative, that did not follow this general behaviour (Trend 3) probably due to a different mechanism of interaction (as compound **20**).

Finally, coumarin **43**, which could be considered the cyclic derivative of **4**, was unable to inhibit the biofilm growth, confirming the essential role of the side chain.

As for the anti-biofilm performance, some additional considerations could be drawn. Generally, ZA derivatives followed the same behaviors described for the anti-biofilm activity, with following exceptions: i) derivatives **5** and **6**, which have a methoxy group and a fluorine atom in the *para* position on the phenyl ring respectively, showed an anti-biofilm performance slightly lower than that of the cinnamic acid **4**; ii) the anti-biofilm performance of compound **9** turned out to be similar to the corresponding analogue **17**, in which the chlorine atom was at position 2 on the phenyl ring. However, the general trend, according to which the substitution at position 2 was not well tolerated, was confirmed; iii) the anti-biofilm performance of the disubstituted halogen derivatives (**15**, **16**, **19**) was in general lower than that of the monosubstituted counterparts as well as the anti-biofilm activity. The same was not observed for compounds (**20**, **22**), which were characterized by electron donating substituents, whose monosubstituted analogues showed a decreased anti-biofilm performance; iv) the esters **26** and **28** showed the opposite behaviour, since the methyl ester **26** exhibited an anti-biofilm performance lower than that of the ethyl ester **28**; v) the majority of *cis* derivatives (**32–38**) were found to have a global anti-biofilm performance promoting biofilm formation.

Our data clearly indicate that: i) the *para*-sulfoxy ester group is not necessary for exploiting the anti-biofilm activity of the molecule, but rather the cinnamic acid scaffold is responsible for the anti-biofilm performance (condition 1); ii) the anti-biofilm activity of ZA derivatives depends upon the presence of a carboxylate anion and consequently by its hydrogen-donating ability. Basically, the acidity (and thus the ability to donate hydrogen) of ZA derivatives depends on the nature of electron-donating (EDG) and electron-withdrawing (EWG) groups on the phenyl ring. EDG increases the charge density of the carboxylate anion and therefore decreases acidity. Conversely, EWG withdraws negative charge from the carboxylate anion and thus increases acidity (condition 2); iii) the conjugated aromatic system is instrumental for the anti-biofilm activities of ZA and its analogues since the presence of the double bond stabilized the carboxylate anion (condition 3).

As for condition 1, [26] hypothesized that the antifouling effect of ZA lies in the high affinity for water shown by its sulfate group which increases the cell surface hydrophility. In contrast, Villa *et al.* [16] demonstrated that the activity of methyl zosterate, in which the carboxylate moiety was replaced by a methyl ester, had no anti-biofilm ability, suggesting that the activity of ZA sodium salt is not due to the presence of the sulfate ester as confirmed by the present study. It is likely that the sulfate ester makes the active cinnamic moiety more soluble and less toxic. Phytochemical investigation revealed that in several seagrass species sulfotransferase enzymes catalyze the sulfation of a wide range of compounds, including phenolic acid compounds [27, 28]. Sulfoconjugation converts compounds into more water soluble metabolites than a non-sulfated molecule. Thus it is more mobile in the water-based sap of the vascular transport system during their translocation from local leaf to systemic leaves. As a

consequence, ZA facilitated excretion from the plant and a more rapidly local and systemic plant response against the pathogen attack [29]. Moreover, sulfonated compounds are generally less toxic to plant cells than non-sulfonated forms. In fact cinnamic acids are rarely found free in plants but generally in the less toxic form of esters [30]. Cinnamic acid is reported to be a potent auxin-inhibitor, affecting both the development of root hairs and phloem tissues and thus limiting the conduction of water, minerals and nutrients, which lead to growth reduction and changes in morphology of plants [31]. Indeed, the addition of a sulfoxy group to cinnamic active moiety is a strategy that plants exert to protect themself from the toxic effect of the active molecule [29].

In line with the abovementioned conditions 2 and 3, an overall view of the obtained results highlighted that compounds typically bearing a carboxylate anion in a conjugated system, showed the best anti-biofilm activity and were effective at sublethal concentrations. Furthermore, derivatives with para substituents (**1**–**3**, **5**–**12**) on the phenyl ring displayed different degrees of *E*. *coli* inhibition growth. Since the strength of a carboxylic acid depends mainly on the extent of its ionization, analogues **7** and **12**, with strong EWG on the phenyl ring (nitro and cyano groups, respectively), reduced the electron density of the oxygen atoms, leading to loss of activity. On the contrary, compounds bearing moderate and strong EDG (compounds **2** and **5**, respectively methyl and methoxy derivatives) enhance the negative charge and exhibited good values of inhibition. Moreover, our results demonstrated that compounds in which the conformation of the double bond was changed (**4** *vs* **37**, *trans*/*cis* isomers), as well as saturated derivatives (**5** *vs* **39**, unsaturated/saturated derivatives) decreased the ability to inhibit biofilm formation. Thus, the more the electron density of the carboxylic group of the ZA derivatives increases, the more effective the molecules are as anti-biofilm compounds at sublethal concentrations.

Indeed, our work highlighted the structural determinants for the ZA anti-biofilm activity, and provided the prospects to generate more potent derivatives based on the same ZA active scaffold and with a better anti-biofilm activity compared to ZA.

The ZA active scaffold is functional in protein interaction

Assuming that primary events triggering the processes related to the anti-biofilm activity relied on an interaction with a protein, the whole *E*. *coli* soluble proteome was screened in order to evaluate the functional ability of the anti-biofilm ZA active scaffold in targeting a protein. To this purpose, a pull-down system combined with mass spectrometry-based approach was developed. The pull-down approach consisted essentially of: i) a batch incubation with the protein extract to favour protein binding to the ZA active scaffold grafted on a solid support; ii) column-packing of the incubated solid support in a gravity-flow chromatographic system and extensive washing of the unbound material; iii) elution of bound protein.

The results obtained from the library screening were instrumental for the application of the affinity pull-down approach. In fact, structure-activity relationship data are necessary to immobilize the anti-biofilm compound on an affinity matrix in a site-selective manner, without compromising the biological activity of the molecule under investigation.

Taking into account the results from the library analysis, we considered: i) the cinnamic acid scaffold responsible for the anti-biofilm performance of ZA (ZA active scaffold), and ii) the *para* position on the phenyl ring as a good anchoring point for matrix immobilisation. Thus, the *p*-aminocinnamic acid (*p*-ACA; **10**) was chosen as the proper molecule to covalently bound via an amide bond the ZA active scaffold to a sepharose matrix bearing 6-aminohexanoic spacer arms activated by *N*-hydroxysuccinimide esterification (*p*-ACA/matrix).

Starting from the evidence that *p*-ACA shows a good fluorescent signal (Fig 8A), fluorescence analysis was carried out on suspensions of the sepharose matrix in order to monitor the effect of the coupling reaction. The *p*-ACA/matrix suspension showed fluorescence, whereas no fluorescence was detectable in suspensions of the control matrix (EA/matrix) prepared by omitting *p*-ACA in the coupling reaction (Fig 8B). Emission and excitation maxima were 426 nm and 350 nm for the *p*-ACA/matrix suspension, and 443 nm and 380 nm for the *p*-ACA solution. Considering that fluorescence of free *p*-ACA was conserved after the immobilisation and that the shift of emission wavelength can be due likely to the different conditions of the samples (suspension *vs* solution), fluorescence data strongly suggest that the coupling reaction did not alter the fluorogenic structure of the *p*-aminocinnamate group.

In order to verify the presence of the grafted ZA active scaffold, *p*-ACA/matrix was hydrolysed and the mixture was submitted to thin layer chromatography (TLC) analysis. A TLC spot co-migrating with *p*-ACA was exhibited (data not shown). The hydrolysate was also analyzed by mass spectrometric techniques. Mass spectrometry analysis registered a monoisotopic single charged mass ($[M+H]^+$) of 164.069 *m/z* in the hydrolysates produced by *p*-ACA/matrix (Fig 9). The theoretical $[M+H]^+$ for *p*-ACA is reported to be 164.071 *m/z*. There is significant evidence that *p*-ACA was released during hydrolysis (experimental mass error of 0.002 Da) and consequently *p*-ACA/matrix was successfully functionalized with the ZA active scaffold bound in the *para* position via the hydrolysable amide.

The pull-down approach was carried out with soluble protein extract from *E. coli*, and protein material bound to the *p*-ACA/matrix was eluted by competition with sodium cinnamate or sodium zosterate. Collected fractions were analysed by SDS-PAGE (Fig 10). One main band at $M_r \sim 25000$ was always observable in the SDS-PAGE profiles of fractions eluted using either sodium cinnamate or sodium zosterate as competitors in independent pull-down experiments. No further bands were observable in the fractions collected when NaCl concentration was increased up to 1 M after the end of the elution by competition. Moreover no bands were eluted when the control matrix (EA/matrix) replaced *p*-ACA/matrix in the pull-down approach. According to an estimation based on the band intensity, ~10 μg were pull-down from 90 mg soluble protein extracted from 2.5 g cell wet pellet (500 mL *E. coli* culture). All together the results from the SDS-PAGE analysis showed that i) the immobilized ZA active scaffold is functional in protein binding, ii) mainly a single protein ($M_r \sim 25000$) from the *E. coli* soluble protein extract is bound, and iii) the bound protein is released by competition with either ZA or cinnamic acid.

The proteins pulled-down from the *p*-ACA/matrix by competition with either cinnamic acid or ZA and resolved by SDS-PAGE were excised from the gel and submitted to mass spectrometric analysis for protein identification. The identified proteins and their characteristics are reported in Table 1 and S2 Table. In all cases mass spectrometric analysis gave back a result with an high score and coverage percentage, suggesting an unequivocal identification of the protein. Mass spectrometric analysis revealed that NADH:quinone dehydrogenase (WrbA; accession number: P0A8G6) was pulled-down by competition with either sodium cinnamate or sodium zosterate, thus showing that the ZA active scaffold is functional in the interaction with a specific protein from the *E. coli* soluble extract.

WrbA is an FMN-dependent flavoprotein bearing NADH:quinone oxidoreductase (NQO) activity [32]. Crystallographic studies reported that WrbA is characterized by a tetrameric quaternary structure with a hydrophobic active site pocket that provides an ideal stacking environment for aromatic moieties [33]. FAD-dependent homodimeric NQOs (e.g. human and rat NQO1) are inhibited by certain coumarins and flavones (e.g. dicoumarol, warfarin, esculetin, phenindione, chrysin, curcumin) [34–36]. The crystal structure of the human Nqo1 in complex with dicoumarol was reported, and it was shown that one of the coumarin rings of dicoumarol

Cmpd	Total value
37	6
24	5
35	5
38	4
18	3
29	3
30	3
34	3
40	3
15	2
25	2
27	2
39	2
42	2
43	2
19	1
26	1
31	1
33	1
36	1
7	0
12	0
14	0
32	0
16	-1
5	-2
6	-2
13	-2
21	-2
23	-2
28	-2
ZA	-3
2	-3
4	-3
9	-3
10	-3
17	-3
22	-4
1	-5
11	-5
20	-6
41	-6
8	-8
3	-9

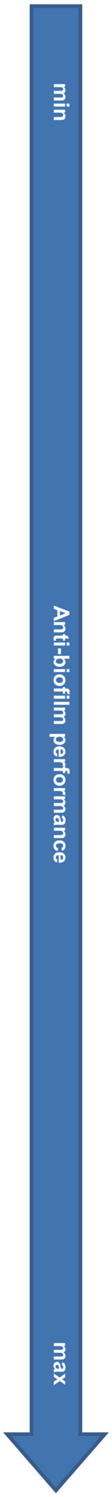

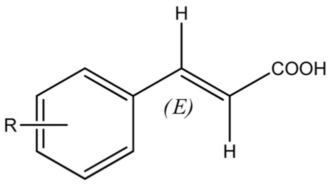

R=4-OSO_3H (**ZA**); R=4-OH (**1**); R=4-CH_3 (**2**);

R=4-CHO (**3**); R=H (**4**); R=4-COOH (**8**); R=4-Cl (**9**);

R=4-NH_2 (**10**); R=4-CF_3 (**11**); R=2-Cl (**17**);

R=3,4-OH (**20**); R=3-OH,4-NH_2 (**22**).

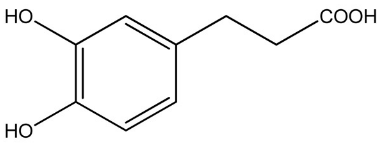

41

Fig 8. Fluorescence analysis of matrix functionalization. A) Emission spectrum (λ_{exc} 380 nm) of 5 mM *p*-ACA in 0.4 M $NaHCO_3$, 1 M NaCl (pH 8,3). B) Emission spectra (λ_{exc} 350 nm) of suspensions of *p*-ACA/matrix (50 µl, drained volume; solid line) and EA/matrix (50 µl, drained volume; dashed line) in 2 mL of 0.4 M $NaHCO_3$, 1 M NaCl (pH 8,3). A. U., arbitrary units.

doi:10.1371/journal.pone.0131519.g008

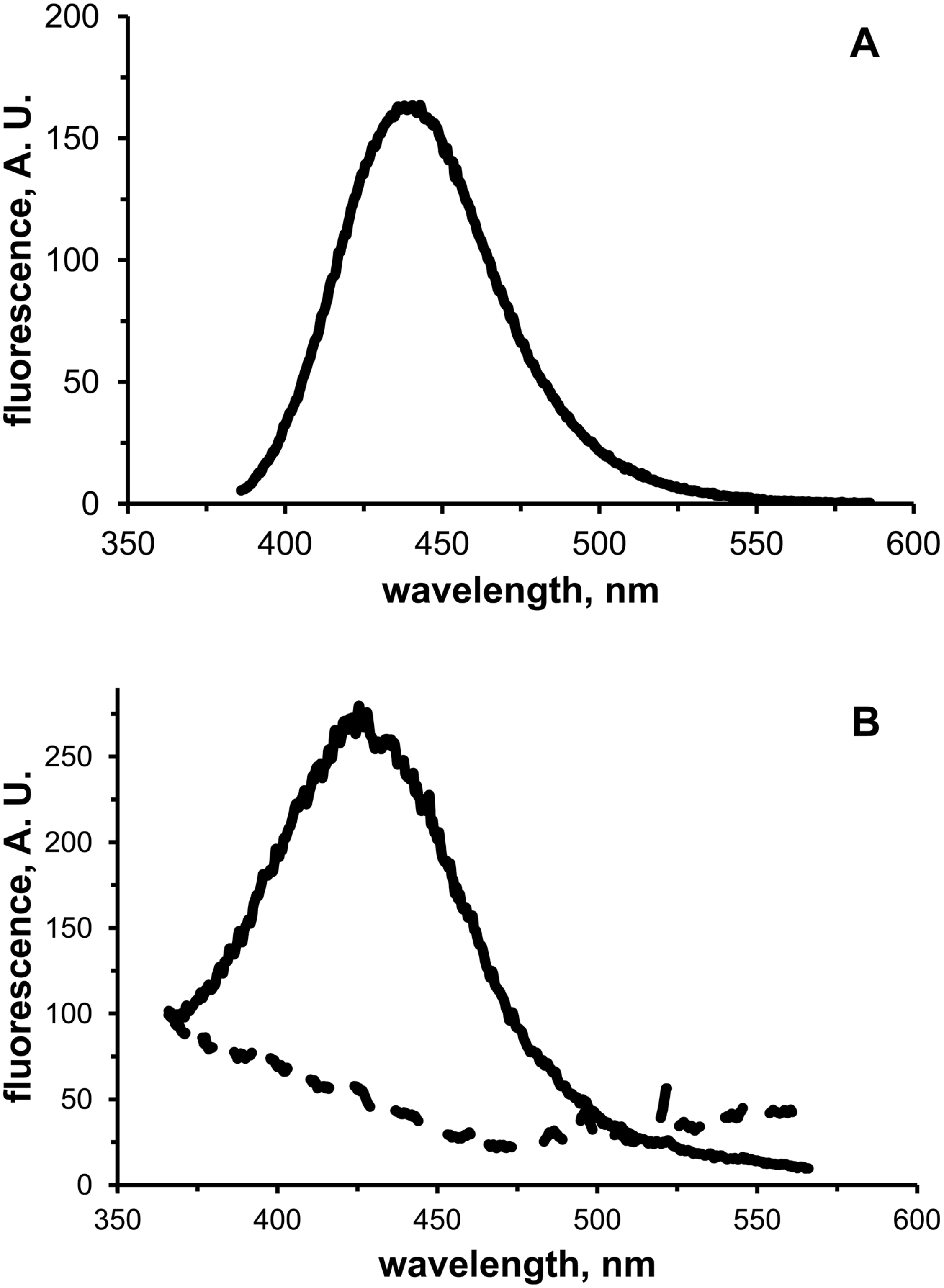

Fig 9. Mass spectrometry analysis of hydrolyzed *p*-ACA/matrix. The eXtracted Ion Current (XIC) profile at 164.069 *m/z*, recorded during the LC-MS run, is shown. LC retention time on main peak is evidenced. The 164.069 *m/z* ion corresponds (ΔM = 0.002 Da) to the theoretical *m/z* value of the $[M+H]^+$ of *p*-ACA. NL: normalization level.

doi:10.1371/journal.pone.0131519.g009

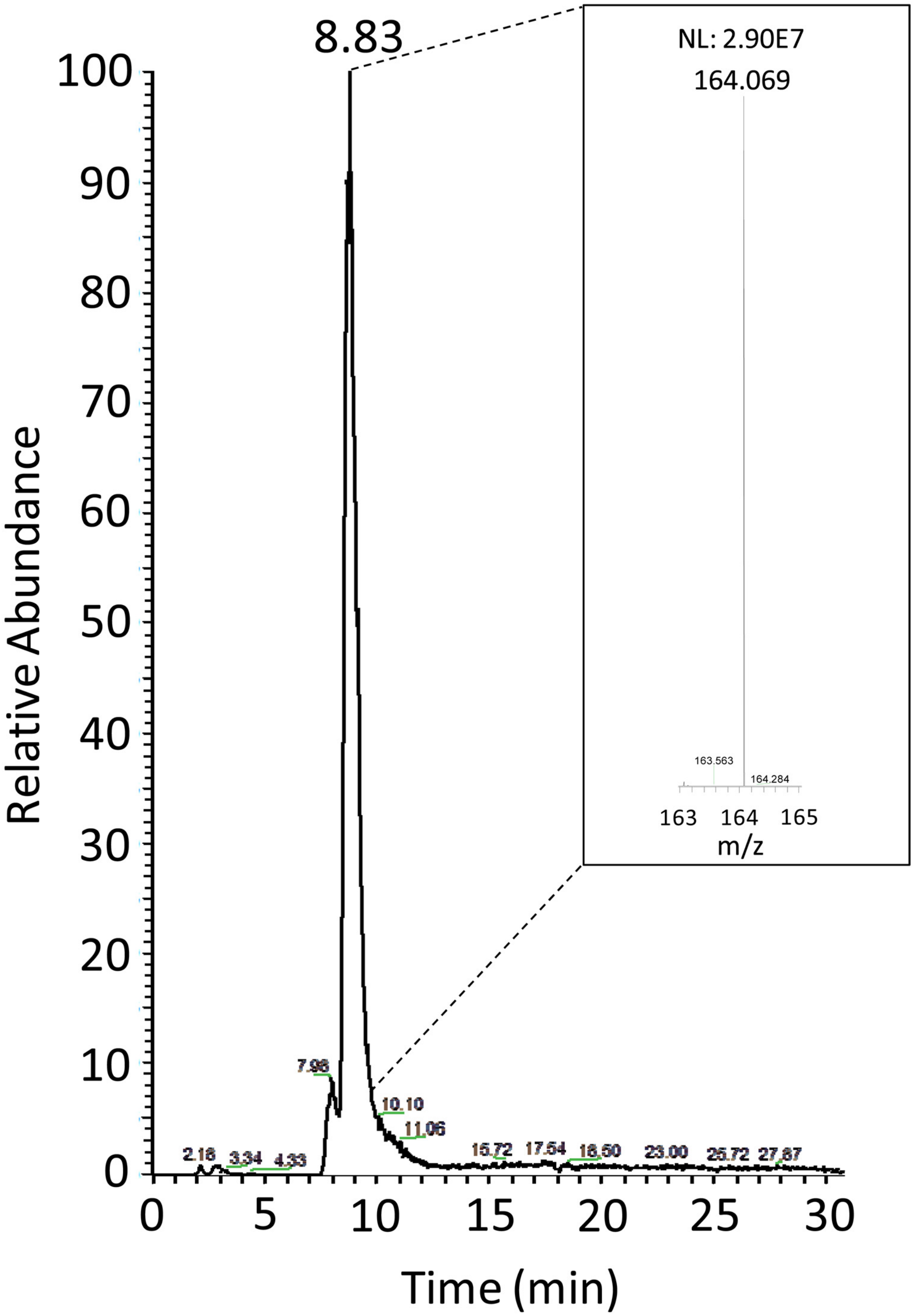

Fig 10. SDS-PAGE analysis of protein pull-down. Coomassie Blue stained SDS-PAGEs of representative protein-pull-down experiments carried out using sodium cinnamate (CA) or sodium zosterate (ZA) as elution competitors and matrixes functionalized (*p*-ACA/matrix) or not (EA/matrix) with the ZA active scaffold are reported. CE, crude extract (35 μg); U, unbound fraction (35 μg); W_1, W_2, washing step fractions; E_1-E_3, elution step fractions. The W and E samples were TCA-precipitated from 870 μL fraction aliquots. The M_rs and positions of marker proteins are reported on left side.

doi:10.1371/journal.pone.0131519.g010

Table 1. Mass spectrometry protein identification.

Comp[a]	Accession[b]	Gene[c]	Protein[d]	Score[e]	Coverage[f]	# Peptide[g]	# PSM[h]	MW (KDa)[i]	Aas[k]
CA	P0A8G6	*wrbA*	NAD(P)H quinone dehydrogenase	189.78	70.20	11	85	20.8	198
ZA	P0A8G6	*wrbA*	NAD(P)H quinone dehydrogenase	235.56	89.90	15	93	20.8	198

a) competitor used in the elution step;

b) alphanumeric unique protein sequence identifier provided by UniProtKB/Swiss-Prot protein knowledge base;

c) gene name;

d) protein name;

e) protein identification's SEQUEST HT Score;

f) percentage of protein sequence covered by identified peptides;

g) total number of identified peptide sequences (peptide spectrum matches);

h) number of identified peptides matching to the protein;

i) theoretically calculated molecular weight;

k) number of amino acids.

doi:10.1371/journal.pone.0131519.t001

was stacked parallel to the isoalloxazine ring of FAD in the active site [37]. The crystal structure of *E. coli* WrbA with oxidized FMN bound reveals a close relationship to the mammalian Nqo1 [38]. Indeed it appears that the NQO active site is well adapted to accommodate phenolic compounds such as ZA and cinnamic acid, suggesting that interaction of the ZA active scaffold with WrbA may involve the active site function.

The transcription factor CsgD (curlin subunit gene D) from *E. coli* is considered the master regulator of the biofilm formation. The *wrbA* gene was shown to be one of the regulation targets of CsgD and the distribution of the CgsD-binding regions in the gene suggests that the expression of *wrbA* is upregulated by CgsD supporting a WrbA role in biofilm formation [39].

Moreover, the *wrbA* gene expression was recognized to be, among few other stress-related genes, under the regulon of the *rpoS*-encoded alternative sigma factor σ^S [40, 41]. The *rpoS* gene appears to be directly involved in the biosynthesis of signal molecules such as the tryptophan metabolite indole which has recently been proved to participate in various aspects of bacterial life including virulence induction, cell cycle regulation, stress resistance, genetic stability, control of metabolic feedback and it is also proposed to be a quorum sensing signal with an important role in the biofilm formation process [42, 43]. Indole is generated by the cytoplasmic enzyme, tryptophanase, which hydrolyses tryptophan to produce indole, pyruvate and ammonia, a reaction that occurs exclusively in bacteria. In *E. coli*, this process is catalysed by TnaA [44]. In particular, RpoS appears to promote tryptophan degradation by inducing the tryptophanase gene *tnaA*, which results in the production of indole, and by stimulating expression of the WrbA protein, which might strengthen negative regulation of the *trp* biosynthetic operon by an unknown mechanism [41, 45]. The *E. coli* WrbA (W, i.e. tryptophan, Repressor Binding protein) was reported to promote complex formations between the tryptophan repressor TrpR and its DNA operator sequences [40]. Subsequent experiments addressed the influence of WrbA on the TrpR-DNA complex, and as these did not show any effect, the involvement of WrbA in transcription regulation was revoked [46]. Although a physical connection to the tryptophan repressor was actually retracted, a physiological connection between WrbA and TrpR cannot be ruled out [46]. It is reported that either too little or too much indole can abolish the ability to increase biofilm formation, in a dose dependent manner and with strain specific activity [42]. In a previous study [19], it was shown that *E. coli*, grown in the presence of ZA, exhibited enhanced tryptophanase activity and an increased amount of indole. It can be

hypothesized that the ZA active scaffold interacts with WrbA with subsequent consequences on the indole production.

WrbA has been reported to be involved in maintaining quinones in a fully reduced state [32]. Quinones are compounds generally tethered to the plasma membrane or freely traversing the lipid bilayer. Although these compounds are essential for normal electron transport by cycling between the oxidized (hydroquinone) state and the two-electron reduced (hydroquinol) state, it has been demonstrated that such quinonoids also participate in deleterious redox cycling through direct interactions with single-electron acceptors such as O_2, leading to the accumulation of reactive oxygen species (ROS) such as superoxide, hydrogen peroxide, and the hydroxyl radical responsible for a devastating consequence in the cells [47]. In order to guard against the production of ROS from one-electron redox cycling, cells have evolved NQOs to maintain quinones in a reduced state as a measure of protection against oxidative stress. A previous study [19] demonstrated that ZA makes *E. coli* cells more prone to accumulate ROS leading to a stress condition, to which the bacterium responds by activating defensive mechanisms against oxidative damage. We can hypothesize that the ZA active scaffold may negatively modulate the oxidoreductase activity of WrbA leading to a ROS accumulation inside cells affecting biofilm formation. Recent data proved the ability of ROS to modulate quorum sensing and showed their important role in biofilm formation [48, 49]. The upset of the ROS level caused by the ZA active scaffold might interfere with the quorum sensing signal destabilizing the biofilm formation. Interestingly, it was suggested that ROS play a role in modulating the indole signaling pathway by the induction of the TnaA expression [50]. Kuczyńska-Wisnik *et al.* [51] demonstrated that an *E. coli* mutant strain that experiences endogenous oxidative stress shows an enhanced expression of tryptophanase and an increased indole production that delays the biofilm formation. In addition, antibiotics that promote ROS formation inhibit development of *E. coli* biofilm in an indole-dependent way, which means that bacteria also use indole signals to modulate pathways dealing with the oxidative stress, and resulting in biofilm repression [52].

A BLAST search against all non-redundant databases of Bacteria using WrbA as the query protein sequence returned 5792 sequences with identity between 40% and 100% to WrbA (expect-value threshold $\leq 10^{-5}$), most of which belonged to different *E. coli* strains, *Shigella* ssp., *Enterobacter* ssp., *Salmonella* ssp., *Klebsiella* ssp., *Cronobacter* ssp., *Yersinia* ssp., *Serratia* ssp., *Burkholderia* ssp. and *Pseudomonas* ssp.. A similar search inside Fungi databases returned 490 sequences with identity between 36% and 91% to WrbA (expect-value threshold $\leq 10^{-5}$) from many species, including *Candida* spp., *Clavispora* spp., *Aspergillus* spp. and *Fusarium* spp.. The BLAST search revealed that WrbA sequences are widespread suggesting an important role of this protein in the microorganism's life, possibly associated to the response against environmental stresses. The presence of WrbA-like proteins in other microorganisms, including those involved in human infections or agricultural diseases, may suppose that ZA active scaffold derivatives could interact with these proteins leading to a possible effect on the biofilm development of these microorganisms. In this direction, it was demonstrated that ZA is able to significantly reduce *Pseudomonas putida* biofilm [18] and *Candida albicans* biofilm [17].

Thus this part of the work highlighted the functional role of the ZA active scaffold in the interaction with WrbA and, on the basis of the above considerations, an altered production of both indole and ROS can be envisaged as the consequence of the interaction, affecting the biofilm formation.

We envision that the findings gained from this work will be used to covalently couple the anti-biofilm molecules to a supporting material in such a way as to deliver the active moiety to the cellular target. In this approach, no molecule would be leached from the surface, thus sidestepping the problem of the compound kinetics release, providing long-term protection against bacterial colonization, and reducing the risk of developing resistant microbial strains, as the

concentration of the anti-biofilm agent would be constantly below the lethal concentration while maintaining a bioactive molecule.

Conclusion

In this study, the bottlenecks limiting the exploitation of ZA as a preventive strategy to counteract biofilm formation, were successfully explored and overcome. The screening of the 43-member library of small molecules based on ZA scaffold against *E. coli* biofilm highlighted the key role of cinnamic scaffold as specific structural feature responsible for the ZA anti-biofilm performance. Moreover structure-anti-biofilm activity relationship considerations revealed that the anti-biofilm activity of ZA derivatives depended upon the presence of a carboxylate anion and, consequently, on its hydrogen-donating ability. In addition, the conjugated aromatic system was instrumental to the anti-biofilm activities of ZA and its analogues since the presence of the double bond stabilized the carboxylate anion. Therefore this study has evidenced that the cinnamic acid scaffold can be considered a potential template for a class of promising anti-biofilm compounds.

The evaluation of the functional ability of the ZA active scaffold to interact with a protein led to the discovery of WrbA as a molecular target involved in ZA anti-biofilm activity, suggesting a possible role of this protein in the biofilm formation process. However, it could be possible that there is a mechanism triggered by ZA interaction with this protein, sofurther clarification studies are necessary. The confirmation of a relation between biofilm formation and WrbA would open the way for the development of new and more effective therapies against biofilm formation.

Supporting Information

S1 Protocol. General procedure for the synthesis of compounds 2, 7, 8, 11, 17, 23, 24, 33, 34, 36–38.
(PDF)

S1 Table. Planktonic growth and cell adhesion in the presence of ZA and compounds 1–43. Percentage reduction respect to the negative control is calculated as (ZA-related compound data—control data) x 100 / control data. a) Percentage reduction of the maximum specific growth rate: code (0) $x > -10\%$; code (- 1)- $10\% \leq x < -20\%$; code (- 2)- $20\% \leq x < -30\%$; code (- 3) $x \leq -30\%$; b) Percentage reduction of the number of adhered cells: code (+ 1) $x > +20\%$; code (0) $+20\% \leq x < -20\%$; code (- 1)- $20\% \leq x < -30\%$; code (- 2)- $30\% \leq x < -40\%$; code (- 3) $x \leq -40\%$. According to post hoc analysis (Tukey's HSD, $p < 0.05$), data sharing the same letter indicate no significant difference.
(PDF)

S2 Table. Mass spectrometry peptide sequencing.
(PDF)

Acknowledgments

We wish to thank Dr. F. Iavarone (Institute of Biochemistry and Clinical Biochemistry, Catholic University of Rome, Italy) for assistance in mass spectrometry experiments, and Federica Larocca for assistance in the pull-down experiments.

Author Contributions

Conceived and designed the experiments: FF FV AG FC. Performed the experiments: CC SDO AV VM SB. Analyzed the data: CC SDO FV SV AG AV VM FF FC. Contributed reagents/

materials/analysis tools: FC SV FF. Wrote the paper: CC SDO SV FV FF. Designed the experiments: CC SV SB.

References

1. Costerton JW, Cheng KJ, Geesey GG, Ladd TI, Nickel JC, Dasgupta M, et al. Bacterial biofilms in nature and disease. Annu Rev Microbiol. 1987; 41: 435–464. PMID: 3318676
2. Von Eiff C, Kohnen W, Becker K, Jansen B. Modern strategies in the prevention of implant-associated infections. Int J Artif Organs. 2005; 28: 1146–1156. PMID: 16353121
3. Hall-Stoodley L, Costerton WJ, Stoodley P. Bacterial biofilms: from the natural environment to infectious diseases. Nat Rev Microbiol. 2004; 2: 95–108. PMID: 15040259
4. Smith AW. Biofilms and antibiotic therapy: Is there a role for combating bacterial resistance by the use of novel drug delivery systems? Adv Drug Delivery Rev. 2005; 57: 1539–1550.
5. Darouiche RO. Antimicrobial coating of devices for prevention of infection: principles and protection. Int J Artif Organs. 2007; 30: 820–827. PMID: 17918128
6. Donlan RM. Biofilm elimination on intravascular catheters: important considerations for the infectious disease practitioner. Clin Infect Dis. 2011; 52: 1038–1045. doi: 10.1093/cid/cir077 PMID: 21460321
7. Andersson DI, Hughes D. Antibiotic resistance and its cost: is it possible to reverse resistance? Nat Rev Microbiol. 2010; 8: 260–271. doi: 10.1038/nrmicro2319 PMID: 20208551
8. Directive 98/8/EC of the European Parliament and of the Council of 16 February 1998 concerning the placing of biocidal products on the market. Available: http://eurlex.europa.eu/LexUriServ/site/en/consleg/1998/L/01998L000820070119-en.pdf.
9. SCENIHR. The scientific committee on emerging and newly identified health risks report. 2009. Available: http://ec.europa.eu/health/opinions/en/biocides-antibiotic-resistance/l-3/8-riskassessment.htm.
10. European Food Safety Authority and European Centre for Disease Prevention and Control. The European Union summary report on antimicrobial resistance in zoonotic and indicator bacteria from humans, animals and food in 2010. EFSA J 10:2598. Available: www.efsa.europa.eu/efsajournal.
11. Villa F, Villa S, Gelain A, Cappitelli F. Sublethal activity of small molecules from natural sources and their synthetic derivatives against biofilm forming nosocomial pathogens. Curr Top Med Chem. 2013; 13: 3184–3204. PMID: 24200356
12. Villa F, Giacomucci L, Polo A, Principi P, Toniolo L, Levi M, et al. N-vanillylnonanamide tested as a non-toxic antifoulant, applied to surfaces in a polyurethane coating. Biotechnol Lett. 2009; 31: 1407–1413. doi: 10.1007/s10529-009-0031-4 PMID: 19488677
13. Villa F, Borgonovo G, Cappitelli F, Giussani B, Bassoli A. Sublethal concentrations of *Muscari comosum* bulb extract suppress adhesion and induce detachment of sessile yeast cells. Biofouling. 2012; 28: 1107–1117. doi: 10.1080/08927014.2012.734811 PMID: 23061484
14. Cappitelli F, Polo A, Villa F. Biofilm formation in food processing environments is still poorly understood and controlled. Food Eng Rev. 2014; 6: 29–42.
15. Rasko DA, Sperandio V. Anti-virulence strategies to combat bacteria-mediated disease. Nat Rev Drug Discovery. 2010; 9: 117–128.
16. Villa F, Albanese D, Giussani B, Stewart P, Daffonchio D, Cappitelli F. Hindering biofilm formation with zosteric acid. Biofouling. 2010; 26: 739–752. doi: 10.1080/08927014.2010.511197 PMID: 20711895
17. Villa F, Pitts B, Stewart PS, Giussani B, Roncoroni S, Albanese D, et al. Efficacy of zosteric acid sodium salt on the yeast biofilm model *Candida albicans*. Microbial Ecol. 2011; 62: 584–598.
18. Polo A, Foladori P, Ponti B, Bettinetti R, Gambino M, Villa F, et al. Evaluation of zosteric acid for mitigating biofilm formation of *Pseudomonas putida* isolated from a membrane bioreactor system. Int J Mol Sci. 2014; 15: 9497–9518. doi: 10.3390/ijms15069497 PMID: 24879523
19. Villa F, Remelli W, Forlani F, Vitali A, Cappitelli F. Altered expression level of *Escherichia coli* proteins in response to treatment with the antifouling agent zosteric acid sodium salt. Environ Microbiol. 2012; 14: 1753–1761. doi: 10.1111/j.1462-2920.2011.02678.x PMID: 22176949
20. Szymanski W, Wu B, Weiner B, de Wildeman S, Feringa BL, Janssen DB. Phenylalanine aminomutase-catalyzed addition of ammonia to substituted cinnamic acids: a route to enantiopure alpha- and beta-amino acids. J Org Chem. 2009; 74: 9152–9157. doi: 10.1021/jo901833y PMID: 19894731
21. De P, Koumba Yoya G, Constant P, Bedos-Belval F, Duran H, Saffon N, et al. Design, synthesis, and biological evaluation of new cinnamic derivatives as antituberculosis agents. J Med Chem. 2011; 54: 1449–1461. doi: 10.1021/jm101510d PMID: 21309577
22. Zwietering MH, Jongenburger I, Rombouts FM, van't Riet K. Modeling of the bacterial growth curve. Appl Environ Microbiol. 1990; 56: 1875–1881. PMID: 16348228

23. Bradford MM. Rapid and sensitive method for the quantitation of microgram quantities of protein utilizing the principle of protein-dye binding. Anal Biochem. 1976; 72: 248–254. PMID: 942051

24. Laemmli UK. Cleavage of structural proteins during the assembly of the head of bacteriophage T4. Nature. 1970; 227: 680–685. PMID: 5432063

25. Di Pasqua R, Mamone G, Ferranti P, Ercolini D, Mauriello G. Changes in the proteome of *Salmonella enterica* serovar Thompson as stress adaptation to sublethal concentrations of thymol. Proteomics. 2010; 10: 1040–1049. doi: 10.1002/pmic.200900568 PMID: 20049861

26. Stanley MS, Callow ME, Perry R, Alberte RS, Smith R, Callow JA. Inhibition of fungal spore adhesion by zosteric acid as the basis for a novel, non-toxic crop protection technology. Phytopathology. 2002; 92: 378–383. doi: 10.1094/PHYTO.2002.92.4.378 PMID: 18942950

27. Papenbrock J. Highlights in seagrasses' phylogeny, physiology, and metabolism: what makes them special?. ISRN Botany. 2012;ID 103892.

28. Hirschmann F, Krause F, Papenbrock J. The multi-protein family of sulfotransferases in plants: composition, occurrence, substrate specificity, and functions. Front Plant Sci. 2014; 5: 556. doi: 10.3389/fpls.2014.00556 PMID: 25360143

29. Baek D, Pathange P, Chung J-S, Jiang J, Gao L, Oikawa A, et al. A stress-inducible sulphotransferase sulphonates salicylic acid and confers pathogen resistance in Arabidopsis. Plant Cell Environ. 2010; 33: 1383–1392. doi: 10.1111/j.1365-3040.2010.02156.x PMID: 20374532

30. Giada MDLR. Food phenolic compounds: main classes, sources and their antioxidant power. In: Morales-González JA, editor. Oxidative stress and chronic degenerative diseases—A role for antioxidants. InTech; 2013. pp. 87–112.

31. Jitareanu A, Boz I, Tataringa G, Zbancioc AM, Stanescu U. The effects of some cinnamic acid derivatives on the architecture of *Phaseolus vulgaris* roots. Rom Biotech Lett. 2013; 18: 8317–8326.

32. Patridge EV., Ferry JG. WrbA from *Escherichia coli* and *Archaeoglobus fulgidus* is an NAD(P)H: quinone oxidoreductase. J Bacteriol. 2006; 188: 3498–3506. PMID: 16672604

33. Andrade SLA, Patridge EV, Ferry JG, Einsle O. Crystal Structure of the NADH:Quinone Oxidoreductase WrbA from *Escherichia coli*. J Bacteriol. 2007; 189: 9101–9107. PMID: 17951395

34. Hosoda S, Nakamura W, Hayashi K. Properties and reaction mechanism of DT diaphorase from rat liver. J Biol Chem. 1974; 249: 6416–6423. PMID: 4138437

35. Garten S, Wosilait WD. Comparative study of the binding of coumarin anticoagulants and serum albumins. Biochem Pharmacol. 1971; 20: 1661–1668. PMID: 4126702

36. Tsvetkov P, Asher G, Reiss V, Shaul Y, Sachs L, Lotem J. Inhibition of NAD(P)H:quinone oxidoreductase 1 activity and induction of p53 degradation by the natural phenolic compound curcumin. Proc Natl Acad Sci USA. 2005; 102: 5535–5540. PMID: 15809436

37. Asher G, Dym O, Tsvetkov P, Adler J, Shaul Y. The crystal structure of NAD(P)H quinone oxidoreductase 1 in complex with its potent inhibitor dicoumarol. Biochemistry. 2006; 45: 6372–6378. PMID: 16700548

38. Carey J, Brynda J, Wolfová J, Grandori R, Gustavsson T, Ettrich R, et al. WrbA bridges bacterial flavodoxins and eukaryotic NAD(P)H:quinone oxidoreductases. Protein Sci. 2007; 16: 2301–2305. PMID: 17893367

39. Ogasawara H, Yamamoto K, Ishihama A. Role of the biofilm master regulator CsgD in cross-regulation between biofilm formation and flagellar synthesis. J Bacteriol. 2011; 193: 2587–2597. doi: 10.1128/JB.01468-10 PMID: 21421764

40. Yang W, Ni L, Somerville RL. A stationary-phase protein of *Escherichia coli* that affects the mode of association between the Trp repressor protein and operator-bearing DNA. Proc Natl Acad Sci USA. 1993; 90: 5796–5800. PMID: 8516330

41. Lacour S, Landini P. σS-Dependent gene expression at the onset of stationary phase in Escherichia coli: function of σs-dependent genes and identification of their promoter sequences. J Bateriol. 2004; 186: 7186–7195.

42. Hu M, Zhang C, Mu Y, Shen Q, Feng Y. Indole Affects Biofilm Formation in Bacteria. Indian J Microbiol. 2010; 50: 362–368. doi: 10.1007/s12088-011-0142-1 PMID: 22282601

43. Worthington RJ, Richards JJ, Melander C. Small molecule control of bacterial biofilms. Org Biomol Chem. 2012; 10: 7457–7474. doi: 10.1039/c2ob25835h PMID: 22733439

44. Li G, Young KD. Indole production by the tryptophanase TnaA in *Escherichia coli* is determined by the amount of exogenous tryptophan. Microbiology, 2013; 159: 402–410. doi: 10.1099/mic.0.064139-0 PMID: 23397453

45. Collet A, Vilain S, Cosette P, Junter GA, Jouenne T, Phillips RS, et al. Protein expression in Escherichia coli S17-1 biofilms: impact of indole. A Van Leeuw J Microb. 2007; 91: 71–85.

46. Grandori R, Khalifah P, Boice JA, Fairman R, Giovanielli K, Carey J. Biochemical characterization of WrbA, founding member of a new family of multimeric flavodoxin-like proteins. J Biol Chem. 1998; 273: 20960–20966 PMID: 9694845

47. Adams MA, Jia Z. Structural and biochemical evidence for an enzymatic quinone redox cycle in *Escherichia coli*: identification of a novel quinol monooxygenase. J Biol Chem. 2005; 280: 8358–8363. PMID: 15613473

48. Čáp M, Váchová L, Palková Z. Reactive oxygen species in the signaling and adaptation of multicellular microbial communities. Oxid Med Cell Longev. 2012;ID 976753.

49. Villa F, Remelli W, Forlani F, Gambino M, Landini P, Cappitelli F. Effects of chronic sublethal oxidative stress on *Azotobacter vinelandii* biofilm formation. Biofouling. 2012; 28: 823–833. doi: 10.1080/08927014.2012.715285 PMID: 22871137

50. Ren D, Bedzyk LA, Thomas SM, Ye RW, Wood TK. Gene expression in *Escherichia coli* biofilms. Appl Microbiol Biotechnol. 2004; 64: 515–524. PMID: 14727089

51. Kuczynska-Wisnik D, Matuszewska E, Laskowska E. *Escherichia coli* heat-shock proteins IbpA and IbpB affect biofilm formation by influencing the level of extracellular indole. Microbiology. 2010; 156: 148–157. doi: 10.1099/mic.0.032334-0 PMID: 19797360

52. Kuczyńska-Wisnik D, Matuszewska E, Furmanek-Blaszk B, Leszczyńska D, Grudowska A, Szczepaniak P, et al. Antibiotics promoting oxidative stress inhibit formation of *Escherichia coli* biofilm via indole signalling. Res Microbiol. 2010; 161: 847–853. doi: 10.1016/j.resmic.2010.09.012 PMID: 20868745
